# Discovery and Genome Characterization of Three New Rhabdoviruses Infecting *Passiflora* spp. in Brazil

**DOI:** 10.3390/v17050725

**Published:** 2025-05-19

**Authors:** Andreza Henrique Vidal, Ana Clara Rodrigues Abreu, Jorge Flávio Sousa Dantas-Filho, Monique Jacob Xavier Vianna, Cristiano Lacorte, Emanuel Felipe Medeiros Abreu, Gustavo Pereira Felix, Dione Mendes Teixeira Alves-Freitas, Bruna Pinheiro-Lima, Isadora Nogueira, Fabio Gelape Faleiro, Raul Castro Carriello Rosa, Onildo Nunes Jesus, Marcio Martinello Sanches, Yam Sousa Santos, Rosana Blawid, José Leonardo Santos Jiménez, Maite Freitas Silva Vaslin, Elliot Watanabe Kitajima, Magnolia de Araujo Campos, Rafaela Salgado Fontenele, Arvind Varsani, Fernando Lucas Melo, Simone Graça Ribeiro

**Affiliations:** 1 Embrapa Recursos Genéticos e Biotecnologia, Brasília 70770-917, DF, Brazil; 2 PPG BIOMOL, Instituto de Biologia, Universidade de Brasília, Brasília 70910-900, DF, Brazil; 3 Instituto de Biologia, Universidade de Brasília, Brasília 70910-900, DF, Brazil; 4 Embrapa Cerrados, Planaltina 73310-970, DF, Brazil; 5 Embrapa Agrobiologia, Seropédica 23891-000, RJ, Brazil; 6 Embrapa Mandioca e Fruticultura, Cruz das Almas 44380-000, BA, Brazil; 7 Embrapa Gado de Corte, Campo Grande 79106-550, MS, Brazil; 8 Departamento de Microbiologia, Instituto de Biotecnologia Aplicada à Agropecuária, Universidade Federal de Viçosa (UFV), Viçosa 36570-900, MG, Brazil; 9 Departamento de Agronomia, Universidade Federal Rural de Pernambuco, Recife 52171-900, PE, Brazil; 10 Departamento de Virologia, Instituto de Microbiologia Paulo de Góes, Universidade Federal do Rio de Janeiro, Rio de Janeiro 21941-902, RJ, Brazil; 11 Departamento de Fitopatologia, Escola Superior de Agricultura Luiz de Queiroz, Piracicaba 13418-900, SP, Brazil; 12 Centro de Educação e Saúde, Universidade Federal de Campina Grande, Cuité 58175-000, PB, Brazil; 13 The Biodesign Center for Fundamental and Applied Microbiomics, School of Life Sciences, Center for Evolution and Medicine, Arizona State University, Tempe, AZ 85287, USA; 14 Onsite Genomics, Brasília 70610-433, DF, Brazil

**Keywords:** plant rhabdovirus, high-throughput sequencing, virus particles, microscopy, gammacytorhabdovirus, alphanucleorhabdovirus

## Abstract

This study aimed to explore the RNA viruses affecting *Passiflora* species in Brazil. Our results enhance the understanding of the viruses that infect *Passiflora* plants by identifying and characterizing three previously unrecognized viruses: Passiflora cytorhabdovirus (PFCV), Passiflora nucleorhabdovirus 1 (PaNV1), and Passiflora nucleorhabdovirus 2 (PaNV2). These rhabdoviruses were identified through high-throughput sequencing and validated by reverse transcription-polymerase chain reaction (RT-PCR) in various *Passiflora* species. PFCV has a genome organization 3′-N-P-P3-P4-M-G-P7-L-5′ and was classified as a novel member of the *Gammacytorhabdovirus* genus. A particularly noteworthy feature of PFCV is its glycoprotein, as the genomes of other gammarhabdoviruses do not contain this gene. PFCV has a high incidence across multiple locations and was identified in plants from Northeastern, Central, and Southeastern Brazil. PaNV1 with genome structure 3′-N-P-P3-M-G-L-5′ and PaNV2 with genome organization 3′-N-X-P-Y-M-G-L-5′ are new members of the *Alphanucleorhabdovirus* genus and have a more restricted occurrence. Importantly, all three viruses were found in mixed infections alongside at least one other virus. In situ observations confirmed mixed infections, with PaNV2 particles co-located in tissues with a potyvirus and a carlavirus. Phylogenetic and glycoprotein sequence similarity network analysis provided insights into their evolutionary placement and potential vector associations. These findings expand the known diversity of rhabdoviruses in *Passiflora* and contribute to the understanding of their evolution and epidemiology.

## 1. Introduction

Recent research has substantially increased the knowledge about the diversity of viruses that infect important crops worldwide. The greater use of high-throughput sequencing (HTS) technologies has facilitated these advances, allowing for the identification of viruses in different environments, regardless of pathology, and without prior knowledge of the pathogen genome sequences. This approach has been intensely applied in recent years for the identification of viruses in the *Rhabdoviridae* family [1,2,3,4,5,6,7].

Passion fruit is produced by plants of the genus *Passiflora* (Passifloraceae family), cultivated in various regions of the world. Brazil is one of the most extensive centers of origin and diversity of *Passiflora* species [8] and is responsible for the largest passion fruit production in the world, with an estimated ~711 tons produced in 2023 [9]. It is used to generate fresh fruit and is highly valued in the food industry. Passion fruits are extensively used in the cosmetic and herbal medicine industries and have been explored and cultivated as ornamental plants [10].

There are very few reports of rhabdoviruses infecting passion fruit plants worldwide. The first record, dating back to the 1980s, refers to an unclassified nucleorhabdovirus, called passionfruit rhabdovirus (PRV), found infecting *Passiflora edulis* grown in Sydney, Australia [11]. Later, another probable nucleorhabdovirus, called passion fruit vein clearing virus (PVCV), was identified in passion fruit plants across several Brazilian states and was associated with a condition of “stunting” in these plants [12,13,14]. However, no molecular data or taxonomic characterization is associated with PRV and PVCV. Recently, two viruses belonging to the species *Betacytorhabdovirus caricae* have been identified in passion fruit plants. The citrus-associated rhabdovirus (CiaRV) was reported in *P. edulis* in China [3], and the bean-associated cytorhabdovirus (BaCV) was identified in various species of *Passiflora* in Brazil [15]. Additionally, other rhabdoviruses have been identified in transcriptome data, including the betacytorhabdoviruses barley yellow striate mosaic virus (BYSMV, *Betacytorhabdovirus hordei*) in *P. edulis* from China [1], and Passiflora betacytorhabdovirus 1 (PaBCR1, *Betacytorhabdovirus passiflorae*) in *P. caerulea* from China [1,2].

Currently, the family *Rhabdoviridae* is composed of over 500 virus species divided into four subfamilies and 62 genera. The *Betarhabdovirinae* subfamily, comprising the genera *Alphagymnorhavirus*, *Alphanucleorhabdovirus*, *Betagymnorhavirus*, *Betanucleorhabdovirus*, *Alphacytorhabdovirus*, *Betacytorhabdovirus*, *Gammacytorhabdovirus*, *Deltanucleorhabdovirus*, *Dichorhavirus*, *Gammanucleorhabdovirus*, *Trirhavirus,* and *Varicosavirus*, includes viruses that infect plants and arthropod vectors [2,5,16].

Rhabdoviruses have a negative-sense single-stranded RNA genome, with genomes ranging from 10 to 16 kb. Most plant-infecting rhabdoviruses are unsegmented, with a few members having bi-segmented or tri-segmented genomes [2,4,17,18]. The genomic organization of these viruses is generally similar to that of other members of the *Rhabdoviridae* family. They encode at least six conserved canonical genes arranged in the order: 5′ nucleocapsid (N), phosphoprotein (P), matrix protein (M), glycoprotein (G), and RNA-dependent RNA polymerase (L) 3′. However, it is notable that all current members of the genus *Gammacytorhabdovirus* lack the glycoprotein gene [2]. These genes are separated by intergenic regions, which are flanked by relatively conserved sequences responsible for the initiation of mRNA transcription [16]. The genome may also contain additional genes encoding nonstructural accessory proteins flanked by the same conserved intergenic regions that precede the other proteins. In most cases, the functions of the accessory proteins are still unknown [19].

In this study, we used HTS technology to investigate and characterize the RNA viruses of passion fruit plants in Brazil, leading to the identification of new rhabdoviruses associated with this crop.

## 2. Materials and Methods

### 2.1. Plant Material

For HTS sequencing, 59 accessions of passion fruit plants from different species and hybrids were collected in 2017 from the Empresa Brasileira de Pesquisa Agropecuária—EMBRAPA *Passiflora* germplasm bank known as “Banco de Germoplasma ‘Flor da Paixão’—BAG-FP”, located in Planaltina, Distrito Federal, Brazil. In addition, passion fruit samples from mthe state of Bahia were collected in 2015 from open commercial fields. Specifically, *P. edulis* plants were collected in Marcionílio de Souza (*n* = 6), Seabra (*n* = 8), Morro do Chapéu (*n* = 2), Brumado (*n* = 2), Dom Basílio (*n* = 11), Jussiape (*n* = 9), and Lençóis (*n* = 15). In Lençóis, *P. cincinnata* (*n* = 3) and *P. alata (n* = 3) were also collected.

Moreover, additional passion fruit plants, mostly *P. edulis* and *P. alata*, were sampled for RT-PCR evaluation in Paraiba state in 2016 (Cuité, *n* = 24); in Pernambuco state in 2019 (Vitoria de Santo Antão, *n* = 11); and in Rio de Janeiro state in 2021 (Seropédica, *n* = 51). In Distrito Federal, plants were collected from farms in 2017 in Planaltina (*n =* 5); in 2018 in Brazlândia (*n =* 20); and in 2019 in Brasília (*n* = 9), Planaltina (*n* = 14), as well as in an open field at BAG-FP (*Passiflora* spp. *n* = 18).

All leaf samples collected (Table S1) were stored at −80 °C until analysis.

### 2.2. Double-Stranded RNA Extraction, High-Throughput Sequencing, and Bioinformatic Analysis

Double-stranded RNAs (dsRNAs) were extracted from 37 *Passiflora* spp. and hybrid accessions from BAG-FP and organized into two pools (BAG2-PF *n* = 17 plants; BAG3-PF *n* = 20 plants) (Table S1). The dsRNA extraction, dsRNA pools, libraries preparation, and HTS sequencing were conducted according to Vidal et al. [20]. The dsRNA libraries were sequenced using an Illumina HiSeq 2500 sequencer (Macrogen Inc., Seoul, Republic of Korea).

Other passion fruit samples constituted the libraries BAG1-PF (*n* = 21 plants), PM1BA (*n* = 29 plants), and PM2BA (*n* = 30 plants) (Table S1). These libraries were previously sequenced and have been reanalyzed in this study [20,21,22].

For all libraries, paired-end reads (100 bp) generated in the Illumina HiSeq were trimmed and assembled de novo to produce contigs using CLC Genomics Workbench [23]. These de novo assembled contigs were analyzed against a local database of viral sequences using the BLASTx search tool and then validated against the NCBI virus database. The open reading frames (ORFs) were predicted and annotated using ORFfinder (accessible at https://www.ncbi.nlm.nih.gov/orffinder/, accessed on 25 November 2024) and Geneious Prime^®^ 2024.0.4 software [24]. Additionally, BLAST searches (including BLASTn, BLASTx, and BLASTp with the PSI-BLAST algorithm option) were conducted to confirm the obtained sequences in comparison to other sequences available in GenBank (accessible at https://blast.ncbi.nlm.nih.gov/Blast.cgi, accessed on 25 November 2024).

### 2.3. Virus Detection and Sanger Sequence

Based on the virus contigs identified through HTS, virus-specific primers were designed and used in RT-PCR assays to detect viruses in individual samples. Total RNA was isolated from ~100 mg of plant leaf tissue macerated in liquid nitrogen. The extraction was conducted using the TRIzol Reagent (Invitrogen, Carlsbad, CA, USA), following the instructions provided by the manufacturer. RT-PCRs were performed either with the SuperScript™ III One-Step RT-PCR System with Platinum™ Taq DNA Polymerase (Invitrogen, Carlsbad, CA, USA) or the cDNA was synthesized using SuperScript III Reverse Transcriptase (Promega, Madison, WI, USA) with anchored Oligo(dT)20 (50 μM) (Invitrogen, Carlsbad, CA, USA) and random primers (50 μM). The cDNA produced was then used in PCR reactions with specific primers for each DNA fragment.

Two primer sets were employed to detect Passiflora cytorhabdovirus (PFCV). The primers BAG3-PCV7114F/BAG3-PCV8045R amplified a fragment of ~960 nt corresponding to the partial L gene, which was utilized for the initial detection in individual samples. For further confirmation of the identity of the virus by Sanger sequencing, the primer pair PCV-BAG-N-182F/PCV-BAG-N-1648R, which amplifies about 1500 nt covering the complete N and partial P genes, was employed.

Passiflora nucleorhabdovirus 1 (PaNV1) was detected using the primer pair PNV1-GL-7102R/PNV1-L-5779F, which amplifies approximately 1352 nt corresponding to a partial L gene (RNA-directed RNA polymerase).

For Passiflora nucleorhabdovirus 2 (PaNV2), the primers BAG3-PNV4310F/BAG3-PNV5299R, which amplify ~1000 nt corresponding to the partial L gene, were used for the preliminary detection in individual samples. To further confirm the PaNV2’s identity by Sanger sequencing, the primers BAG3-PNV-N-262F/BAG3-PNV-N-1890R, which amplify ~1718 nt corresponding to the complete N and partial X (coding for accessory protein), were used.

All characteristics and sequences of primers used in this study are summarized in Table S2.

All amplicons were visualized by electrophoresis in agarose gel stained with SYBR Safe DNA Gel Stain (Invitrogen, Carlsbad, CA, USA). Amplicons of the expected size from selected samples were excised, gel-purified, and cloned in the CloneJET PCR Cloning vector (Thermo Fisher Scientific, Waltham, MA, USA) following the manufacturer’s instructions. The clones were then Sanger sequenced (Macrogen, Seoul, Republic of Korea). All obtained sequences were assembled and analyzed using Geneious Prime^®^.

### 2.4. Complete Sequence of Rhabdoviruses from Passion Fruit

The PFCV, PaNV1, and PaNV2 genomes from individual plants were recovered by overlapping amplicon sequencing. Primer sets were designed based on PFCV, PaNV1, and PaNV2 contig sequences from HTS, assuring that the produced fragments overlap at least 150 nt (Table S2).

Positive PFCV, PaNV1, and PaNV2 samples were selected to amplify and validate the complete rhabdovirus genomes. The cDNA was synthesized using M-MLV Reverse Transcriptase (Promega, Madison, WI, USA) and subsequently used in PCR assays with Phusion High-Fidelity DNA Polymerase (Thermo Fisher Scientific, Waltham, MA, USA) and virus-specific primers.

To obtain the complete genome of PFCV, various primers were used: PCV-BAG-N-182F/PCV-BAG-N-1648R (~1500 nt, complete N gene), PCV-BAG-NPP3P4-679F/PCV-BAG-NPP3P4-3897R (~3200 nt, partial N gene, complete P and P4 genes), PCV-BAG-P4MG-3660F/PCV-BAG-P4MG-6880R (~3200 nt, par-tial P, complete M and G genes), PCV-BAG-P7L-6676F/PCV-BAG-P7L-9873R (~3200 nt, partial P7 and L genes), PCV-BAG-L-9664F/PCV-BAG-L-11873R (~2200 nt, partial L gene), and PCV-BAG-L-11666F/PCV-L12968R (~1700 nt, partial L gene).

For the recovery of the complete genome of PaNV1, the following the sets of primers were employed: PNV1-NP-119F/PNV1-NP-505F (R) (~2300 nt, complete N gene), PNV1-PG-317F/PNV1-PG-3719R (~3400 nt, complete P and partial G genes), PNV1-GL-3573F/PNV1-GL-7102R (~3500 nt, partial G and L genes), PNV1-L-6944F/PNV1-L-9943R (~3000 nt, partial L gene), and PNV1-L-9668F/PNV1-L-11790R (~2100 nt, partial L gene).

The following sets of primers were used to recover the complete genome of PaNV2: BAG3-PNV-N-262F/BAG3-PNV-N-1890R (~1600 nt, complete N and partial X genes), PNV-BAG-XPYM-1754F/PNV-BAG-XPYM-4979R (~3200 nt, partial X, complete P, and Y, and partial M), PNV-BAG-MGL-4777F/PNV-BAG-MGL-7952R (~3200 nt, partial M, complete G, and partial L genes), PNV-BAG-L-7767F/PNV-BAG-L-11471R (~3700 nt, partial L gene), and PNV-BAG-L-11273F/PNV-BAG-L-13577R (~2300 nt, partial L gene).

The PCR products were visualized on agarose gels, and the appropriate fragments were excised and purified using the Wizard^®^ SV Gel and PCR Clean-Up System kit (Promega, Madison, WI, USA), following the manufacturer’s instructions.

Different strategies were employed for sequencing the amplicons (Figure S1). The PaNV1 genome PCR products were sequenced by primer walking using the Sanger method (Macrogen, Seoul, Republic of Korea). For PaNV2 and PFCV genomes, sequences were generated using Oxford Nanopore Technologies (ONT) with a MinION device.

To prepare the libraries, purified DNAs from each sample were quantified, and approximately 10 ng of each amplicon was pooled together. About 50 ng of DNA was then used to construct the library with the Rapid Barcoding Kit (SQK-RBK004) (Oxford Nanopore Technologies, Oxford, UK) according to the instructions, applying one barcode per sample. Sequencing was carried out in a FLO-MIN106D flow cell (Oxford Nanopore Technologies, Oxford, UK) using a MinION Mk1B sequencer (Oxford Nanopore Technologies, Oxford, UK). The consensus genome was obtained after mapping all sequencing data to the genomes of PFCV and PaNV1 using the Medaka v1.2.3 program (Oxford Nanopore Technologies, Oxford, UK).

The sequences were analyzed and assembled with Geneious Prime^®^ 2024.0.4 [24]. BLASTn, BLASTx, and BLASTp (using the PSI-BLAST algorithm option) searches were performed to check the identities among sequences obtained in this research and other rhabdovirus sequences in GenBank. Open reading frames (ORFs) were predicted and annotated using ORF Finder (accessible at https://www.ncbi.nlm.nih.gov/orffinder/, accessed on 25 November 2024).

### 2.5. 5′ and 3′ End Method for Rapid Amplification of cDNA Ends (RACE)

The RACE method was employed to determine the 5′ and 3′ ends of the rhabdovirus genomes, following the methods outlined by Alves-Freitas et al. [25], Nicolini et al. [26], Schuster et al. [27], and Vidal et al. [21]. Rhabdovirus-specific primers (Table S2) were designed based on the sequence data obtained from HTS and used in the RACE reactions.

For the 3′ RACE, a poly-A tail was first added to the RNA (5 μg) using *Escherichia coli* poly (A) polymerase (New England Biolabs, Ipswich, MA, USA). Subsequently, the cDNA was synthesized with SuperScript™ IV Reverse Transcriptase (Invitrogen, Carlsbad, CA, USA) and the anchored primer M10PacIT50VN (Table S1). The cDNA was then treated with RNase H and RNase A before being used in PCR reactions. The 3′ end fragments were obtained from a single PCR cycle using the cDNA and a combination of M10 and virus-specific gene-specific primers (GSPs): PCV_RACE3c_12839F for PFCV, PNV1_RACE3_11364F for PaNV1, and PNV2_RACE3_13119F for PaNV2 with Phusion High-Fidelity DNA Polymerase (Thermo Fisher Scientific, Waltham, MA, USA).

All PCR products obtained were gel-purified, cloned into pJET 1.2/blunt vector (Thermo Fisher Scientific, Waltham, MA, USA), and Sanger sequenced by primer walking (Macrogen, Seoul, Republic of Korea). The sequences were analyzed using Geneious Prime^®^ 2024.0.4 [24], and the sequences resulting from this analysis were used to design primers required to amplify the 3′ end region of the rhabdoviruses.

The primer PCV_3end_R (Table S2) was designed based on sequences recovered from the RACE 3′ ends and used together with PCV_RACE3c_12839F in PCR assays to confirm the 3′ end of the PFCV isolates. For PaNV1 and PaNV2, the sequences obtained in the 3′ Race reaction were utilized to complete the final genome of the respective viruses.

The 5′ RACE was also performed following the methods described by Alves-Freitas et al. [25], Nicolini et al. [26], Schuster et al. [27], and Vidal et al. [21]. However, the determination of the 5′ end for the three rhabdovirus isolates in this study was unsuccessful despite multiple attempts.

### 2.6. Phylogenetic Analysis

For the phylogenetic analysis, we aligned the complete genomes and the predicted L protein sequences of the viruses in this study with those from species accepted by the International Committee on Taxonomy of Viruses (ICTV) that belong to the genera *Alphacytorhabdovirus*, *Betacytorhabdovirus*, *Gammacytorhabdovirus*, *Alphanucleorhabdovirus*, *Betanucleorhabdovirus*, *Deltanucleorhabdovirus*, and *Gammanucleorhabdovirus*. All sequences were aligned using the MAFFT (v7.490) program [28] with default settings. A maximum-likelihood (ML) phylogenetic tree was inferred from this alignment using IQ-TREE software (v2.3.6) with 1000 ultrafast bootstrap replicates and the “find and apply best model” option [29]. All the sequence accession numbers used to construct the ML tree are listed in Supplementary Tables S6 and S7.

Pairwise identity scores for PFCV, PaNV1, and PaNV2 genes, as well as their predicted proteins, were calculated using the MAFFT alignment program implemented in the Sequence Demarcation Tool (SDT) version 1.2 [30].

The amino acid sequences of the glycoproteins encoded by the rhabdoviruses in this study, along with those available in GenBank (Tables S6 and S7), were used to generate a sequence similarity network. This was done using the Enzyme Function Initiative–Enzyme Similarity Tool (EFI–EST) [31] with a sequence alignment score threshold of 35, and a minimum E-value threshold of 1 × 10^−5^. The glycoproteins network was generated and visualized in Cytoscape v3.10.3 [32].

### 2.7. Transmission Electron Microscopy

Cytopathic effects and virus particles were observed in situ using transmission electron microscopy (TEM). Leaves from sample accessions 1624 (*P. riparia*) and 1626 (*P. gardneri*) from BAG-FP, which tested positive for rhabdovirus, were prepared for electron microscopy examination according to Pinheiro et al. [33]. Ultrathin sections were visualized under a JEOL JEM 1011 transmission electron microscope (JEOL, Tokyo, Japan).

### 2.8. Distribution of PFCV, PaNV1, and PaNV2 in Passion Fruit Plants in Brazil

In this study, we evaluated not only the plant accessions from BAG-FP that were used in the HTS analysis, but also additional passion fruit samples collected from commercial and experimental fields were evaluated for infection with PFCV, PaNV1, and PaNV2 (Table S1). The passion fruit plants were collected from various locations, including Paraiba (Cuité *n* = 24), Pernambuco (Vitoria de Santo Antão *n* = 11), Rio de Janeiro (Seropédica *n* = 51), and in the Distrito Federal (Planaltina *n* = 19, Brasilia *n =* 9, Brazlândia *n* = 20, BAG-PF *n* = 18). These samples were evaluated by RT-PCR.

Some of the plants screened were part of previous studies that investigated other viruses [15,20,21,22] and are stored in our collection at −80 °C.

PCR detections were performed as described previously, using the set of primers PCV-BAG-N-182F/PCV-BAG-N-1648R for PFCV, PNV1-GL-7102R/PNV1-L-5779F for PaNV1, and BAG3-PNV-N-262F/BAG3-PNV-N-1890R for PaNV2 (Table S2).

Additionally, we assessed the presence of other viruses investigated in our previous studies [15,20,21,22]. These viruses include lettuce chlorosis virus (LCV, *Crinivirus lactucachlorosi*, *Closteroviridae*), cowpea aphid-borne mosaic virus (CABMV, *Potyvirus vignae*), bean-associated cytorhabdovirus, cowpea mild mottle virus (CPMMV, *Carlavirus vignae*), and cucurbit aphid-borne yellows virus (CABYV, *Polerovirus CABYV*) [15,20,21,22].

The PCR assays were conducted with the following set of primers: for LCV we used LCVRNA22793F/LCVRNA23997R primers, which amplify the partial RNA2- HSP70h/p6.4/p60 genes of LCV [20]; for amplification of BaCV, primers BaCV-6491F/BaCV-7178R were employed to amplify the partial G/L gene of BaCV [21,25]. For CABMV, we used CABMVLNJP2492F/CABMVLNJP3373R primers, which amplify the partial HC-Pro/p3 genes [20]; to amplify the partial CP/MP genes of CABYV, we utilized the CE-9F and CE-10R primers [22,34]. Two primer sets were utilized to amplify the carlavirus CPMMV. For the screening of the BAG-FP plants, we employed primers CPMMVB22095F/CPMMVB22535R that amplify the partial CP gene [15]. In addition, the passion fruit plants from open fields were tested with CPMMV-4000F/CPMMV-4500R, which amplify the partial RdRp [25,35]. All primer sequences are described in Table S2.

## 3. Results and Discussion

### 3.1. Preliminary Identification of Rhabdoviruses from Passion Fruit Plants in Brazil

The discovery and identification of new and known viruses have significantly increased due to the use of high-throughput sequencing (HTS) technology and bioinformatics tools. In this study, HTS-derived de novo assembled contigs enabled the identification of sequences related to rhabdoviruses in various *Passiflora* species.

Analysis of HTS data from libraries BAG1-PF, BAG2-FP, and BAG3-FP (which are accessions of *Passiflora* species from the “Banco Ativo de Germoplasma Flor da Paixão—BAG-FP), along with PM1BA and PM2BA, derived from passion fruit plants from commercial fields in the state of Bahia, revealed contigs with low nucleotide identity to known rhabdoviruses in the BAG2-FP, BAG3-FP, and PM1BA libraries. Bioinformatics analysis identified partial genomic sequences of putative rhabdoviruses.

A contig named Passiflora cytorhabdovirus (PFCV) and a second contig named Passiflora nucleorhabdovirus 2 (PaNV2) were identified in the BAG3-FP library. Additionally, two further sequences were identified and predicted from the PM1BA library and provisionally named Passiflora nucleorhabdovirus 1 (PaNV1).

The PFCV sequence is 13,548 nt long. A Blastx search showed the highest identity hit at 51.97% (with 43% coverage) matching the L protein of Lupinus gammacytorhabdovirus 1 (LupGammAcV-1, *Gammacytorhabdovirus lupinis*, GenBank accession DBA36915).

PaNV2 is 13,720 nt in length, with BLASTx analysis indicating a highest amino acid identity of 45% (with 42% coverage) with the L protein of the Physostegia chlorotic mottle virus (PhCMoV, *Alphanucleorhabdovirus physostegiae*, GenBank accession QDF44105), a member of the genus *Alphanucleorhabdovirus*.

For PaNV1, two sequences longer than 1000 nt were identified in the PM1BA library data, both showing low identities with members of the genus *Alphanucleorhabdovirus*. The first PaNV1 contig is 1695 nt, corresponding to a partial ORF1/N gene (1347 nt; 449 aa), which shares 40.2% amino acid identity (with 69% coverage) with the N of Artemisia capillaris nucleorhabdovirus 1 (*Alphanucleorhabdovirus artemisiae*, GenBank accession UKL15216). The second PaNV1 contig sequence has 12,062 nt in length, sharing the highest amino acid identity of 52.4% (with 41% coverage) with the L of the papaya nucleorhabdovirus 1 (GenBank accession QHB15169) and the second highest amino acid identity of 39% (with 48% coverage) with the L of ArtCaNV1 (GenBank accession OM372677).

Data analyses indicated that PFCV is not limited to the BAG3 library. Mapping of sequencing reads from all libraries (BAG1-FP, BAG2-FP, PM1BA, and PM2BA) using PFCV as the reference sequence revealed the presence of PFCV in both BAG2-FP and PM1BA libraries. In contrast, no evidence of PNV1 and PNV2 was found in the other libraries.

### 3.2. Prediction of the Genomic Sequence of Bean-Associated Cytorhabdovirus, Cowpea Mild Mottle Virus, and Cowpea Aphid-Borne Mosaic Virus in the BAG2-FP and BAG3-FP Libraries

In addition to the newly identified rhabdovirus sequences, the analysis of HTS data from the BAG2-FP and BAG3-FP libraries (accessions of *Passiflora* spp. from the “Banco Ativo de Germoplasma Flor da Paixão—BAG-FP”) has led to the identification of sequences with 99.51% to 100% nt identity to BaCV. Additionally, we identified contigs with nucleotide identities between 84% and 98.89% to CPMMV sequences and contigs corresponding to CABMV with nucleotide identities ranging from 72.05% to 86.39%. These viruses were previously detected in the same plants through PCR and Sanger sequencing in an earlier study, and no complete sequence from passion fruit isolates for BaCV and CPMMV has been determined [15].

The plants identified as positive for BaCV, CPMMV, and CABMV in a previous study [15], along with other samples from BAG-FP, are part of BAG2-FP and BAG3-FP libraries sequenced in this study (Table S1). We performed de novo assembly of contigs and mapped the reads to a reference genome for the three viruses, resulting in the recovery of full and partial genomes.

In the BAG3-FP library, we obtained a consensus sequence of 13422 nt corresponding to BaCV, which was designated as BaCV-BAG3. The genome (Figure S2) displays a typical BaCV genomic organization, containing seven ORFs arranged as 5′-N-P-P3-P4-M-G-L-3′ [25]. The 5′ and 3′ non-coding regions are 121 nt and 142 nt long, respectively. However, the 5′ and 3′ termini of BaCV-BAG3 are incomplete, lacking 29 nucleotides at the 5′ end and 16 nucleotides at the 3′ end when compared to the BaCV-BR-GO isolate (GenBank accession MK202584). In the BAG2-FP library, only partial BaCV sequences were predicted. Blast analysis revealed that BaCV-BAG3 shares nucleotide identities ranging from 78% to 99.77% when compared to other BaCV isolates (GenBank accession MK202584) from Brazil, papaya virus E (PpVE, GenBank accession MH282832) from Ecuador, and CiaRV (GenBank accession MT302541) from China.

Contigs identified with similarity to CPMMV were found in both libraries. A consensus sequence of 8180 nt was generated and designated CPMMV-BAG2. The CPMMV-BAG2 sequence exhibits a typical genomic organization for this virus [36], which includes six ORFs and untranslated regions of 61 nt at the 5′ end and 113 nt at the 3′ end. Compared to CPMMV:BR:GO:14 (GenBank accession MK202583), the 5′ and 3′ ends of the CPMMV-BAG2 are shorter by 11 nucleotides and 5 nucleotides, respectively. The predicted ORFs encode (Figure S2) a replicase, a triple gene block (TGB1, TGB2, and TGB3), the capsid protein (CP), and a nucleic binding protein (NABP) that contains four conserved motifs: methyltransferase, C23 Peptidase, RNA helicase and RNA-dependent RNA polymerase (RdRp) [36]. BLASTn search indicated that the CPMMV-BAG2 sequence has nucleotide identities ranging from 73.27% to 98.81%, with various CPMMV isolates sequences available in GenBank.

The complete genome sequence of CABMV was determined from data collected from the BAG2-PF library. In contrast, only partial sequences of CABMV were identified in the BAG3-FP library. The complete viral genome, named CABMV-BAG2, is 9913 nucleotides long, excluding the poly(A) tail (Figure S2). This full genome is characteristic of potyviruses and contains a large ORF of 9162 nt, which encodes a polyprotein of 3053 amino acids. The genome has all the expected features of a CABMV genome organization, including an encoded polyprotein which, after being cleaved, produces viral proteins 5′-P1-HC_Pro-P3-Pipo-6K1-CI-6K2-NIa_VPg-NIa_Pro-NIb-CP-3′ [37]. The 5′ and 3′ ends of the sequence are complete, containing 136 nt and 228 nt, respectively, in comparison to CABMV/BJL1 (GenBank accession MN124782). BLASTn searches showed that the CABMV-BAG2 sequence has nucleotide identities ranging from 81.87%% to 91.20%, with various CABMV isolates available in GenBank.

The reanalysis of the library BAG1-PF did not lead to the detection of any additional viruses. Only a few short contigs from the RNA1 and RNA2 genomes of LCV and some partial contigs of CABMV were identified, aligning with the findings previously reported by Vidal et al. [20].

The complete genomes of CPMMV-BAG2, CABMV-BAG2, and BaCV-BAG3 have been deposited in the GenBank database under accession numbers PV460962, PV460963, and PV460964, respectively.

### 3.3. Full Genome and Characterization of Rhabdoviruses from Passion Fruit

The low identity of PFCV, PaNV1, and PaNV2 sequences identified in this study with other plant-infecting rhabdoviruses suggests the existence of three new rhabdovirus species infecting *Passiflora* spp. in Brazil. To confirm this hypothesis and molecularly characterize the new rhabdoviruses found in passion fruit, we recovered the complete genomes of the three viruses from individual plants.

RT-PCR assays using primers based on sequences predicted by HTS and the 3′ RACE method allowed the recovery of nearly complete genomes of the three rhabdoviruses identified in this study. The sequences obtained encompass the entire coding region and the complete 3′ end. However, the 5′ ends of the sequences are incomplete because the 5′ RACE method used for these viruses was unsuccessful.

**Figure 1:**
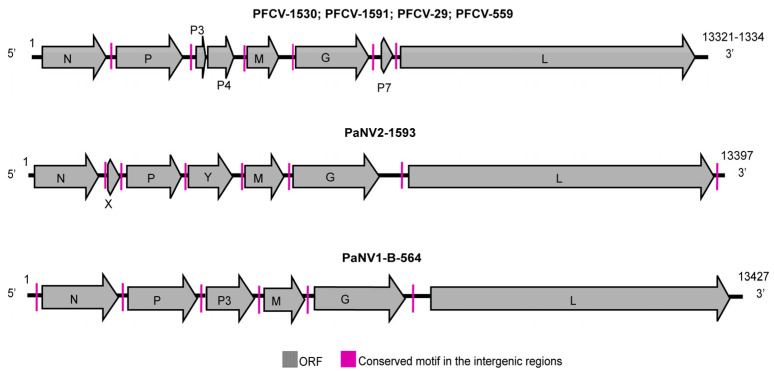
Genome organization of the new rhabdoviruses infecting *Passiflora* species depicting the canonical genes: nucleoprotein (N), phosphoprotein (P), matrix protein (M), glycoprotein (G), and RNA-dependent RNA polymerase—RdRP (L), and non-canonical ORFs encoding P3, P4, P7, X, Y. The rhabdovirus isolates were obtained from different passion fruit species, including PFCV-1530: *P. galbana* and PFCV-1591: *P. eichleriana* x *P. gibertii* from BAG-FP, PFCV-29: *P. edulis* from Rio de Janeiro, and PFCV-559: *P. edulis* from Bahia. PaNV2-1593: *P. galbana* from BAG-FP, and PaNV1-B-564: *P. edulis* from Bahia.

We utilized various sequencing technologies and bioinformatic tools to identify and characterize the diversity of viruses in passion fruit plants in Brazil. We combined Illumina technology for sequencing *Passiflora* pools with Sanger and OTN (MiniION) sequencing methods to sequence the genomes of rhabdovirus isolates. In this way, we could confirm the genome sequences of the new rhabdoviruses infecting passion fruit plants (Figure 1).

#### 3.3.1. Passiflora Cytorhabdovirus (PFCV)

Four passion fruit samples from different regions were selected to obtain the full genome of PFCV. The isolates included PFCV-1530 from *P. galbana* and PFCV-1591 from the *P. eichleriana* x *P. gibertii* hybrid, both collected in BAG-FP, Distrito Federal. Additionally, two more isolates of *P. edulis* were obtained: PFCV-29 from Rio de Janeiro and PFCV-559 from Bahia. All PFCV isolates were sequenced using ONT technology (Figure S1), and the sequences were deposited in the GenBank database under the accession numbers PV460965 to PV460968.

ONT technology, using the MinION sequencer, exhibits versatility in plant virus detection and surveillance, utilizing a portable sequencer and flexible data analysis [38,39]. Here, we used this strategy to sequence the rhabdoviruses in a single run in a fast process without additional primer walking costs, which is commonly necessary for sequencing large genomes. Viruses such as Odontoglossum ringspot virus (*Tobamovirus odontoglossi)*, Cymbidium mosaic virus (*Potexvirus cymbidii*), and Nerine latent virus (*Carlavirus latensnerini*) [40], jasmine virus C (a putative member of genus *Carlavirus, Betaflexiviridae*) [41], Citrus tristeza virus (*Closterovirus tristezae*) [42] are recent examples of plant viruses sequenced using nanopore sequencing technology.

All PFCV isolates (Figure 1) exhibited genomic organization 5′-ORF1/N (1311 nt; 437 aa)—ORF2/P (942 nt; 314 aa)—ORF3/P3 (381 nt; 127 aa)—ORF4/P4 (663 nt; 221 aa)—ORF5/M (597 nt; 199 aa)—ORF6/G (1767 nt; 589 aa)—ORF7/P7 (192 nt; 64 aa)—ORF8/L (6204 nt; 2068 aa)-3′, with some minor differences in genome size (size ranged from 13321 nt to 13334 nt). Additionally, the sizes of the ORF4/P4 gene varied, with PFCV-29 showing a size of 660 nt (220 aa) and the L gene with sizes ranging from 6186 nt to 6204 nt (2062 aa to 2068 aa) among isolates. A conserved 5′-TAAGAAAAMYHW(-/C)(-/M)WGA-3′ consensus intergenic regions separates the genes for the PFCV isolates. The putative proteins encoded by the P3 and P7 genes do not exhibit any similarity to other proteins in the NCBI database. In contrast, the putative P4 protein shares 30.52% amino acid identity (with 96% coverage) with the accessory P3 from Cuscuta gammacytorhabdovirus 1 (CusGCRV1, *Gammacytorhabdovirus alphacuscutae*, GenBank accession DBA36867). Furthermore, P3, P4, and P7 are preceded by the same repetitive region found before the other ORFs, suggesting that they may code for accessory genes (Figure 1). The presence of accessory genes and proteins is a common feature among plant rhabdoviruses [43]. Other cytorhabdoviruses, such as Euphorbia alphacytorhabdovirus 1 (*Alphacytorhabdovirus euphorbiae*), Sesamum betacytorhabdovirus 1 (*Betacytorhabdovirus sesami*) [2], BaCV [25], and yerba mate virus A (*Betacytorhabdovirus yerbamate*) [44], also present putative ORFs named P3 and P4, with the same genomic arrangement as PFCV.

Historically, the genus *Cytorhabdovirus* comprises virus members whose replication and morphogenesis occur in the cytoplasm of infected cells. Phylogenetic trees derived from complete L protein sequences have shown these viruses to form a monophyletic group [16]. Recently, over 90 new cytorhabdoviruses were discovered, and their phylogenetic studies provided support for splitting the genus *Cytorhabdovirus* into three new genera (Table S3): *Alphacytorhabdovirus*, *Betacytorhabdovirus,* and *Gammacytorhabdovirus* [2].

**Figure 2:**
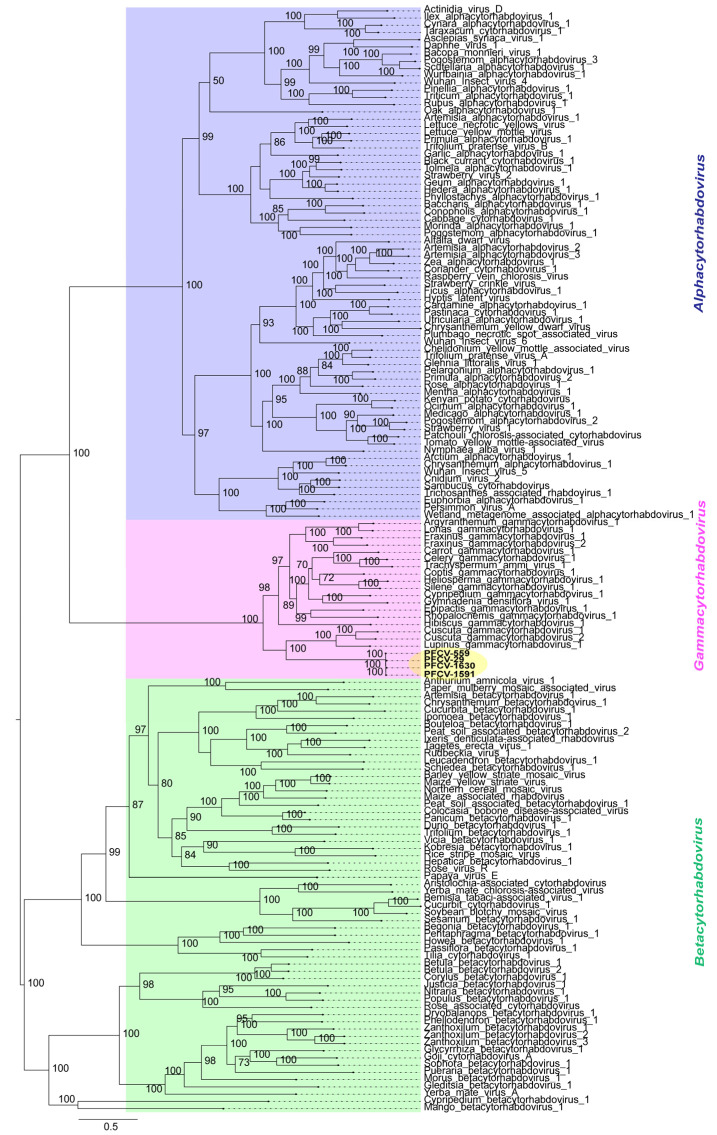
Midpoint-rooted maximum likelihood phylogenetic tree inferred using an amino acid alignment of RdRp (L protein) of Passiflora cytorhabdovirus (PFCV-559; PFCV-29; PFCV-1630; PFCV-1591) isolates with exemplary viruses for the species in the *Alphacytorhabdovirus, Betacytorhabdovirus,* and *Gammacytorhabdovirus* genera. The tree was generated with 1000 ultrafast bootstrap replicates and the “find and apply best model” option. Only the branch support values above 50% are shown. The viruses identified in this study are highlighted in yellow. All the accession numbers used to build the tree are listed in Supplementary Table S3.

To better understand the evolutionary relationships between PFCV and other viruses belonging to the *Alphacytorhabdovirus*, *Betacytorhabdovirus,* and *Gammacytorhabdovirus* genera (Table S3), a phylogenetic tree was constructed based on the deduced L protein amino acid sequences (Figure 2).

The phylogenetic analysis (Figure 2) showed that PFCV isolates grouped within the *Gammacytorhabdovirus* members clade clustering as a sister group of LupGammAcV-1 (GenBank accession BK064358), CusGCRV1 (GenBank accession BK064349), and Cuscuta gammacytorhabdovirus 2 (*Gammacytorhabdovirus betacuscutae,* GenBank accession BK064350).

In terms of pairwise comparisons, the nucleotide and amino acid sequence identities (Table S4) between cognate genes of PFCV isolates and those of exemplar viruses for the species in the genus *Gammacytorhabdovirus* vary significantly. Interestingly, the isolates PFCV-1530 (*P. galbana;* sampled in BAG-FP), PFCV-1591 (*P. eichleriana* x *P. gibertii;* sampled in BAG-FP), PFCV-29 (*P. edulis;* sampled in Rio de Janeiro), and PFCV-559 (*P. edulis*; sampled in Bahia) show considerable similarity, irrespective of the host plant or location. The pairwise comparisons indicated that PFCV isolates share over 99% identity across both their full genomes, as well as in nucleotide and amino acid sequence identities for all cognate genes. However, when comparing PFCV with other gammacytorhabdoviruses, the highest nucleotide identity for the full genome was 40.4% with Fraxinus gammacytorhabdovirus 1 (FraGCRV1, *Gammacytorhabdovirus alphafraxini*, GenBank accession BK064353). In comparison, the identity for canonical genes and predicted protein sequences was less than 43.9% for nucleotides and less than 53.3% for amino acids with all gammacytorhabdoviruses.

The molecular characterization of the four PFCV isolates further substantiates the classification of PFCV as a new species in the *Gammacytorhabdovirus* genus of the *Rhabdoviridae* family. The binomial name “*Gammacytorhabdovirus passionis*” is proposed for PFCV.

Most gammacytorhabdoviruses have been identified through in-silico analysis of plant and fungal transcriptome data [2]. These viruses form a monophyletic group based on phylogenetic trees of the L protein and share a notable characteristic: they lack the G gene. Moreover, Rhopalocnemis gammacytorhabdovirus 1 (*Gammacytorhabdovirus rhopalocnemis,* GenBank accession BK064359) and Epipactis gammacytorhabdovirus 1 (*Gammacytorhabdovirus epipactis*, GenBank accession BK064352) are missing the M protein. Furthermore, FraGCRV1 and Fraxinus gammacytorhabdovirus 2 (FraGCRV2, *Gammacytorhabdovirus betafraxini*, GenBank accession BK064354) contain a small ORF located between the M and L genes [2]. In contrast, all PFCV isolates encode for a glycoprotein and a matrix protein. The glycoprotein is an essential protein in the insect vector-virus interaction [45]. The G protein directly interacts with receptors in the vector cells, induces virus endocytosis, and mediates the fusion of viral and endosomal membranes [16]. Given the phylogenetic relation of gammacytorhabdoviruses, the absence of the G gene, and the identification of FraGCRV1 and FraGCRV2 in a fungal (*Hymenoscyphus fraxineus*) library [2], it was conjectured that this group of viruses might have a fungal vector instead of being transmitted by arthropods [46].

Other viruses in different genera within the *Rhabdoviridae* family and *Betarhabdovirinae* subfamily also lack the G protein. These include Ixeris denticulata-associated rhabdovirus (*Betacytorhabdovirus ixeris*) [47], Tagetes erecta virus 1 (*Betacytorhabdovirus tagetis*) [4], Rudbeckia virus 1 (*Betacytorhabdovirus rudbeckiae*) [48], Cypripedium betacytorhabdovirus 1 (*Betacytorhabdovirus cypripedii*), Hepatica betacytorhabdovirus 1 (*Betacytorhabdovirus hepaticae*), Vicia betacytorhabdovirus 1 (*Betacytorhabdovirus viciae*). Moreover, other viruses have shown irregular patterns in their glycoproteins, as CiaRVs isolates from citrus [3] and patchouli chlorosis-associated cytorhabdovirus (*Alphacytorhabdovirus alphapogostemi*) [49] isolated from patchouli plants. It is speculated that simplification of viral genomes can be an evolutionary advantage, allowing viruses to adapt to plants without relying on arthropod vectors [3]. This is particularly relevant for plants that are propagated vegetatively, such as those identified as hosts of the alpha- and betacytorhabdoviruses mentioned above [2,3,49]. Another possibility is that seeds may transmit viruses that lack the G protein without the mediation of vectors [48].

**Figure 3:**
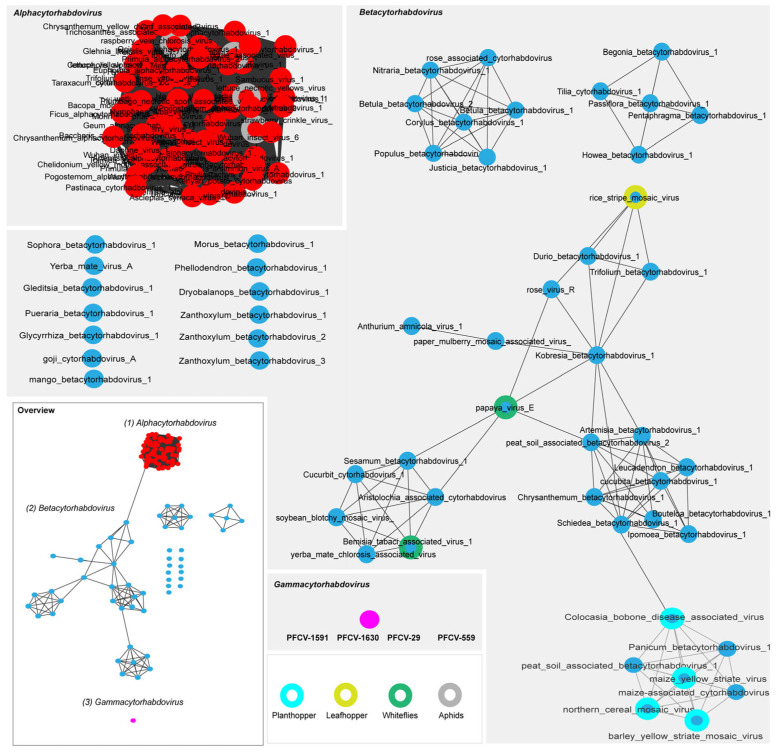
Sequence similarity network analysis of the glycoprotein amino acid (G protein) sequences of Passiflora cytorhabdovirus isolates (PFCV-559; PFCV-29; PFCV-1630; PFCV-1591) and exemplar viruses for the species in the *Alphacytorhabdovirus* and *Betacytorhabdovirus* genera (dataset was created with an amino acid identity cut-off of 90%). Viruses with known insect vectors are highlighted. All the virus sequences accession numbers used are listed in Supplementary Table S3.

Conversely, analyses of G protein also provide invaluable insights into the potential vectors that transmit plant rhabdoviruses [33,50]. Considering that the viral envelope glycoprotein interacts with factors on the surface of vector cells, it is possible that the glycoproteins of plant rhabdoviruses co-evolve with their vectors, similar to what has been proposed for geminivirus capsid proteins and their vectors [51,52]. To investigate this relationship, we performed phylogenetic analyses (Figure S3) and sequence similarity network analyses using the EFI–EST webserver (Figure 3). These analyses utilized the glycoprotein amino acid sequences encoded by members of the *Alphacytorhabdovirus* and *Betacytorhabdovirus* listed in Table S3, as well as PFCV isolates.

Phylogenetic analyses of G proteins (Figure S3) showed that PFCV isolates share a common ancestor with those of alphacytorhabdoviruses, indicating the likely evolutionary connection of these viruses. A similar topology is observed in the phylogenetic tree of the L protein, which includes both gammacytorhabdoviruses and PFCV isolates (Figure 2). This suggests that the viruses more closely related to both gammacytorhabdoviruses and PFCV belong to the *Alphacytorhabdovirus* genus. Consequently, the presence of the G protein in PFCV is a characteristic shared with alphacytorhabdoviruses, while gammacytorhabdoviruses appear to have lost this feature, possibly due to their host plants being propagated vegetatively.

Although passion fruit can be propagated through cuttings, grafting, or tissue culture, sexual propagation through seeds remains the most common method for the passion fruit species grown for food [53]. Therefore, the presence of the G protein raises the possibility that an unidentified insect is involved in the transmission of PFCV.

The sequence similarity network generated with the G protein amino acid sequences (using a >90% identity cut-off) revealed distinct clusters for the viruses belonging to the *Alphacytorhabdovirus* and *Betacytorhabdovirus* genera (Figure 3). This grouping is based on the similarity of their protein sequences and insect vectors. PFCV forms a distinct cluster (seen as a single circle due to the high identity of the G proteins among PFCV isolates), indicating that it is presumably transmitted by an insect vector that has not yet been associated with the other viruses. All *Alphacytorhabdovirus* members were grouped in a unique cluster of highly interconnected proteins. Several viruses known to be aphid-transmitted [54,55,56] were included in this cluster, implying that the other alphacytorhabdoviruses have aphids as vectors. *Betacytorhabdovirus* members are transmitted by various insects, including whiteflies [33], planthoppers [57,58], and leafhoppers [59]. These viruses are grouped into three relational clusters, with some viruses appearing as singletons. Similar clustering was seen previously among BaCV, PpVE, CiaRV, yerba mate chlorosis-associated virus (*Betacytorhabdovirus flaviyerbamate*), cucurbit cytorhabdovirus 1 (*Betacytorhabdovirus alphacucurbitae*), soybean blotchy mosaic virus (*Betacytorhabdovirus glycinis*), Bemisia tabaci associated virus 1 (*Betacytorhabdovirus bemisiae*), and Aristolochia-associated cytorhabdovirus (*Betacytorhabdovirus aristolochiae*), considering the L and G proteins [33,50]. However, experimental transmission by *Bemisia tabaci* has been validated only for BaCV [33] and PpVE [60].

Interestingly, most alpha- and betacytorhabdoviruses were identified and characterized solely through sequencing data [2], meaning that no biological data are associated with them. Our results suggest that some of these viruses share the same vector. While some viruses share an unknown vector, for other viruses, this type of network provides valuable insights into potential insect candidates for virus transmission. For independent viruses in the similarity network, and based on their distance on the phylogenetic tree branches, it appears that, besides their G proteins being quite different, there may be a diversity of undiscovered viruses that could connect these known viruses.

#### 3.3.2. Passiflora Nucleorhabdovirus 1 (PaNV1) and 2 (PaNV2)

The PaNV2 isolate, designated PaNV2-1593, was identified exclusively in sample 1593 (*P. edulis*) in BAG-FP from the Distrito Federal. The isolate was sequenced using ONT technology (Figure S1), and the sequence was deposited in the GenBank database under accession number PV460960.

The genome of PaNV2-1593 (Figure 1) is 13,397 nucleotides long featuring a genomic organization of 5′-ORF1/N (1437 nt; 439 aa)—ORF2/X (321 nt; 107 aa)—ORF3/P (951 nt; 317 aa)—ORF4/Y (867 nt; 289 aa)—ORF5/M (768 nt; 256 aa)—ORF6/G (1824 nt; 608 aa)—ORF7/L (5859 nt; 1593 aa)—3′. The genes are linked by conserved consensus intergenic regions noted as 5′-WHTTARTAAAAACCCWWHMH-3′. The putative X protein encoded by ORF2 does not exhibit similarities to any other viral proteins in GenBank. Meanwhile, the predicted Y protein coded by ORF4 displays 28.07% amino acid identity with 98% coverage with a putative movement protein (Y) of the eggplant mottled dwarf virus (EMDV, *Alphanucleorhabdovirus melongenae*, GenBank accession CDK85636) [61]. These findings suggest the likely presence of a movement protein in PaNV2.

The *Nucleorhabdovirus* genus, which was initially defined by the replication and morphogenesis of the viruses in the cell nucleus, has recently been reclassified into four distinct genera designated as *Alphanucleorhabdovirus, Betanucleorhabdovirus, Deltanucleorhabdovirus,* and *Gammanucleorhabdovirus* (Table S5) [62].

**Figure 4:**
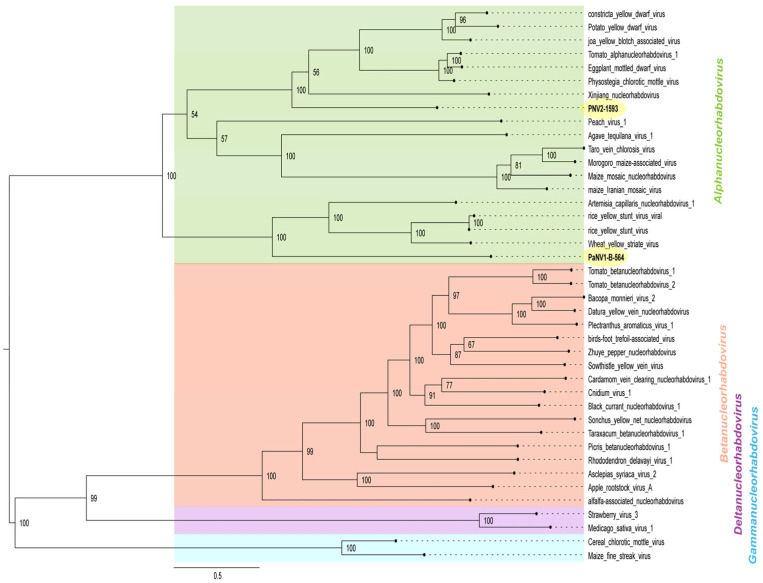
Midpoint-rooted maximum likelihood phylogenetic tree inferred using an amino acid alignment of RdRp (L protein) of Passiflora nucleorhabdovirus 1 (PaNV1-B-564) and Passiflora nucleorhabdovirus 2 (PaNV2-1593) isolates with exemplar viruses for the species in the members of *Alphanucleorhabdovirus*, *Betanucleorhabdovirus*, *Deltanucleorhabdovirus,* and *Gammanucleorhabdovirus* genera. The tree was inferred with 1000 ultrafast bootstrap replicates and the “find and apply best model” option. Only the branch support values above 50% are shown. The viruses identified in this study are highlighted in yellow. All the accession numbers used to construct the tree are listed in Supplementary Table S5.

Phylogenetic analysis based on the L protein amino acid sequences displays clear clusters representing these four genera (Figure 4). PaNV2-1593 grouped with xinjiang nucleorhabdovirus (*Alphanucleorhabdovirus xinjianensis*, GenBank accession MW897039), constricta yellow dwarf virus (CYDV, *Alphanucleorhabdovirus constrictae*, GenBank accession KY549567), potato yellow dwarf virus (PYDV, *Alphanucleorhabdovirus tuberosum*, GenBank accession GU734660), joa yellow blotch associated virus (JYBaV, *Alphanucleorhabdovirus joa*, GenBank accession MW014292), tomato alphanucleorhabdovirus 1 (TARV1, *Alphanucleorhabdovirus lycopersici,* GenBank accession OL472126), PhCMoV (GenBank accession KX636164), and EMDV (GenBank accession KJ082087) (Figure 4).

For the complete genome, pairwise sequence identities between PaNV2-1593 and other alphanucleorhabdoviruses ranged from 32.6% (compared to peach virus 1, *Alphanucleorhabdovirus pruni,* GenBank accession MN520414) to 37.6% (compared to CYDV, GenBank accession KY549567) (Table S6). Nucleotide and amino acid sequence identities between PaNV2-1593 and other alphanucleorhabdoviruses were all below 43.7% and 47.1%, respectively. Furthermore, PaNV2-1593 shares a nucleotide identity of 51.5% with PaNV1-B-564 for the entire genome. When canonical genes and their corresponding amino acid sequences were analyzed, identity values among the passion fruit alphanucleorhabdoviruses ranged from 51.2% to 52.1% at the nucleotide, and from 18.6% to 35.5% at the amino acid (Table S6). These two viruses have substantial divergence in their sequences, which is also reflected in variations in their genomic organization (Figure 1). PaNV1-B-564 isolates code for a putative accessory protein called P3, located between the P and M, while PaNV2 encodes for two accessory proteins, designated X and Y, situated between the N/P and P/M, respectively.

The genomic sequence of PaNV1-B-564 was obtained from a single sample (*P. edulis* sample 564) from Seabra (Table S1) and was determined in this study using Sanger sequencing (Figure S1).

PaNV1-B-564 is 13,427 nucleotides in length and comprises six ORFs arranged as follows: 5′-ORF1/N (1530 nt; 510 aa)-ORF2/P (981 nt; 327 aa)-ORF3/P3 (906 nt; 302 aa)-ORF4/M (828 nt; 276 aa)-ORF5/G (1863 nt; 621 aa)-ORF6/L (5871 nt; 1957 aa)-3′ (Figure 1). Conserved intergenic regions with the consensus sequence 5′-NWRTWWWMAAAACCCYAACYW-3′ connect each gene. In addition, ORF3 of PaNV1-B-564 encodes a P3 accessory protein that does not show similarity to any proteins in GenBank.

Phylogenetic analysis (Figure 4) showed that PaNV1-B-564 clustered with the alphanucleorhabdoviruses ArtCaNV1 (GenBank accession OM372677), wheat yellow striate virus (*Alphanucleorabdovirus tritici*, GenBank accession MG604920), and rice yellow stunt virus (RYSV, *Alphanucleorabdovirus oryzae,* GenBank accession AB011257 and AB516283).

In pairwise nucleotide comparisons (Table S7), the complete genome of PaNV1-B-564 showed the highest identity to PaNV2-1593 (51.5%) and CYDV (41.3%). Nucleotide and amino acid sequence identities were below 42.2% and 41.8%, respectively, when compared to that of other alphanucleorhabdoviruses.

These results indicate that PaNV1 and PaNV2 are distinct viruses that are most closely related to members of the *Alphanucleorabdovirus* genus. Following the species demarcation criteria for *Alphanucleorhabdovirus*, we propose that PaNV1 and PaNV2 represent two novel species within this genus. We suggest the binomial names “*Alphanucleorhabdovirus passionis*” for PaNV1 and “*Alphanucleorhabdovirus passiflorae*” for PaNV2.

**Figure 5:**
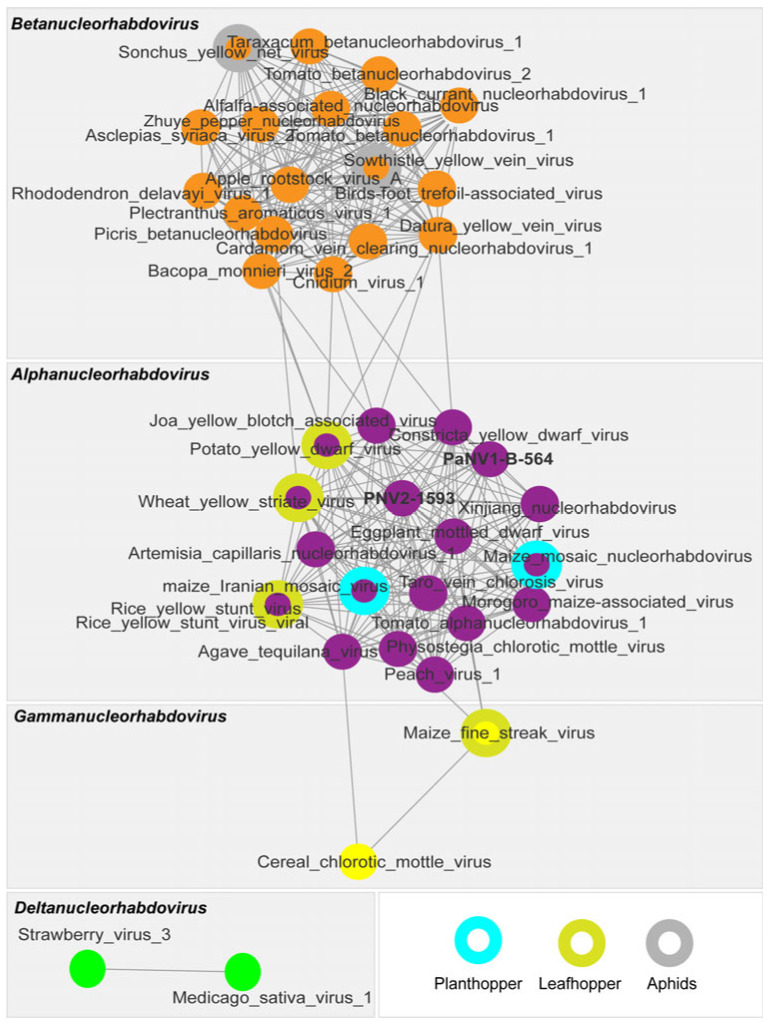
Sequence similarity network analysis of the glycoprotein (G protein) of Passiflora nucleorhabdovirus 1 (PaNV1-B-564), Passiflora nucleorhabdovirus 2 (PaNV2-1593) isolates, and exemplar viruses for the species in the *Alphanucleorhabdovirus*, *Betanucleorhabdovirus*, *Deltanucleorhabdovirus*, and *Gammanucleorhabdovirus* genera (dataset was created with an amino acid identity cut-off of 90%). The viruses that have known insect vectors are highlighted. All the virus sequence accession numbers used in this analysis are listed in Supplementary Table S5.

To gain an insight into the possible insect vectors of PaNV1-B-564 and PaNV2-1593, we performed phylogenetic (Figure S4) and sequence similarity network analyses of glycoprotein amino acid sequences encoded by the *Alphanucleorhabdovirus*, *Betanucleorhabdovirus*, *Deltanucleorhabdovirus*, and *Gammanucleorhabdovirus* listed in Table S5, including PaNV1-B-564 and PaNV2-1593 (Figure 5).

The phylogenetic tree for the G protein (Figure S4) displayed a topology similar to that of the L protein tree (Figure 4), clustering members of the *Alpha-*, *Beta-*, *Delta-*, and *Gammanucleorhabdovirus* genera into four distinct groups. PaNV1-B-564 exhibited closer phylogenetic relationships with ArtCaNV1 (GenBank accession OM372677), RYSV (GenBank accession AB011257 and AB516283), and WYSV (GenBank accession MG604920). In contrast, PaNV2-1593 clustered in a separate clade with TARV1 (GenBank accession OL472126), PhCMoV (GenBank accession KX636164), EMDV (GenBank accession KJ082087), CYDV (GenBank accession KY549567), JYBaV (GenBank accession MW014292), and PYDV (GenBank accession GU734660).

Likewise, similarity network analyses (Figure 5) also revealed distinct clusters for *Alphanucleorhabdovirus*, *Betanucleorhabdovirus*, *Deltanucleorhabdovirus*, and *Gammanucleorhabdovirus* genera, confirming the relationship observed in the G protein phylogenetic tree (Figure 5 and Figure S4). Several of these viruses are known to be transmitted by leafhoppers, planthoppers, or aphids.

Among the members of the *Alphanucleorhabdovirus* genus, RYSV [63], WYSV [64], EMDV, and PYDV [65] are transmitted by leafhoppers, whereas maize mosaic virus (*Alphanucleorhabdovirus maydis*) [66] and maize Iranian mosaic virus (*Alphanucleorhabdovirus zeairanense*) [67] are transmitted by planthoppers. Members of the *Betanucleorhabdovirus* genus, including Sonchus yellow net virus (*Betanucleorhabdovirus retesonchi*) [68] and sowthistle yellow vein virus (*Betanucleorhabdovirus venasonchi*) [69] are transmitted by aphids. The members of the *Deltanucleorhabdovirus* genus, Medicago sativa virus 1 (*Deltanucleorhabdovirus medicagonius*) [70] and strawberry virus 3 (*Deltanucleorhabdovirus fragariae*) [62] have not yet been experimentally associated with a vector. In the *Gammanucleorhabdovirus* genus, maize fine streak virus (*Gammanucleorhabdovirus maydis*) is transmitted by leafhoppers [71].

These results led us to hypothesize that an unidentified leafhopper species may be involved in the transmission of PaNV1 and PaNV2. Leafhoppers like *Empoasca* sp. (green leafhopper) are known to colonize passion fruit [72]. However, experimental data have yet to confirm this suspicion.

Currently, only the rhabdoviruses PRV [11], PVCV [12,13,14], CiaRV [3], BaCV [15], BYSMV [1], and PaBCR1 [1,2] have been reported in *Passiflora* spp. around the world. Among these, PVCV and BaCV have been identified in *Passiflora* plants in Brazil. The three rhabdoviruses characterized in this study—PVCV, PNV1, and PNV2—can now be added to this list, further increasing the known diversity of this group of viruses in passion fruit.

### 3.4. Distribution of the Rhabdoviruses PFCV, PaNV1, PaNV2 in the BAG-FP and Producing Regions of Brazil, and Co-Infection with Other Viruses

Based on PFCV, PaNV1, and PaNV2 sequences, specific primers were designed and employed in RT-PCR assays to detect these rhabdoviruses in individual *Passiflora* samples. Additionally, we evaluated the occurrence of rhabdoviruses in both single and mixed infections involving CABMV, BaCV, CPMMV, LCV, and CABYV in passion fruit plants from various regions of Brazil (Table S1).

PaNV2 was detected in four *Passiflora* accessions (Table S1) from the BAG-FP collection, specifically in *P. galbana* (sample 1593), *P. quadrangularis* (sample 1603), *P. riparia* (sample 1624), and *P. gardneri* (sample 1626). PaNV1 was identified in a single sample, a *P. edulis* plant (sample 564), collected in Seabra, Bahia (Table S1). Both PaNV1 and PaNV2 were initially identified through high-throughput sequencing. Their presence was confirmed by RT-PCR in plants from the Seabra region of Bahia and the BAG-PF collection located in the Distrito Federal, respectively. No evidence of these two viruses was found in samples from other regions (Table S1).

Among the three newly identified rhabdoviruses in this study, PFCV exhibited the highest incidence, being detected in multiple locations across the country. Based on HTS data, PFCV sequences were detected in BAG2, BAG3, and PM1Ba libraries. RT-PCR assays confirmed the presence of PFCV in 17 accessions of the BAG-FP collection, including three positive accessions from the BAG2 library and 14 from the BAG3 library (Table S1). Furthermore, in the PM1BA library, which comprises 29 samples from Bahia, PFCV was detected in 11 plants from various locations. Specifically, the virus was identified in 28.5% (2/7) of plants from Marcionílio de Souza, 37.5% (3/8) from Seabra, 50% (1/2) from Morro do Chapéu, and 45.4% (5/11) from Dom Basílio (Table S1).

In addition to the samples collected from Bahia (BA, Northeastern region) and BAG-FP (DF, Central region), PFCV was identified in several producing regions, including the Distrito Federal (DF, Central region), Rio de Janeiro (RJ, Southeast region), Paraíba (PB, Northeast region), and Pernambuco (PE, Northeast region). This virus infects multiple cultivated, wild, and hybrid *Passiflora* species (Table S1). In the Southeast region, particularly in Seropédica (RJ), PFCV was identified in 94.2% (49/52) of the *P. edulis* plants, including 100% of FB300 genotype plants (25/25) and 88.9% of H09-110/111 hybrids (24/27). This high incidence indicates that the virus is widespread in this area, affecting nearly all evaluated plants. In the Northeast region, PFCV was identified in 9% (1/11) of *P. edulis* plants sampled in Vitória de Santo Antão (PE) and in 16.6% (4/24) of the *P. edulis* plants collected in Cuité (PB).

Additionally, PFCV was detected in 25% (1 *P. edulis* and 4 *P. alata* out of 20) of the plants collected in Brazlândia, in 20% (1 *P. edulis* out of 5) and 71.4% (10 infected *P. edulis* out of 14) of the plants from Planaltina sampled in 2017 and 2019, respectively, and in 5.5% (1 *P. maliformes* out of 18 plants) of the plants maintained in open fields in BAG-FP.

Our results indicate that PFCV is a widely distributed virus, whose occurrence is not restricted to a single Brazilian state. It has been identified in different *Passiflora* species from the accessions maintained in BAG-PF (DF), as well as from cultivated plants in Bahia, Distrito Federal, Paraíba, Pernambuco, and Rio de Janeiro (Table S1). This widespread detection suggests that PFCV is likely present in additional regions across Brazil. These findings also raise the possibility of virus dissemination through infected seedlings or the involvement of an unidentified viral vector.

In previous studies, several viruses were identified in the same plants from BAG-FP and from Bahia and Rio de Janeiro fields, including LCV, CABMV, BaCV, CPMMV, and CABYV [20,21,22,35] (Table S1). Most plants infected with rhabdoviruses were also co-infected with at least one additional virus, primarily CABMV. As shown in Table S1, the presence of these viruses supports previous reports on the frequent occurrence of mixed infections in passion fruit plants [15,20,21,35].

### 3.5. Rhabdovirus Particles

An important characteristic of rhabdoviruses that infect plants is the morphology of the viral particle, as well as the sites of replication and morphogenesis within infected cells. The viral particles are enveloped virions with a bullet-shaped or bacilliform morphology [73].

**Figure 6:**
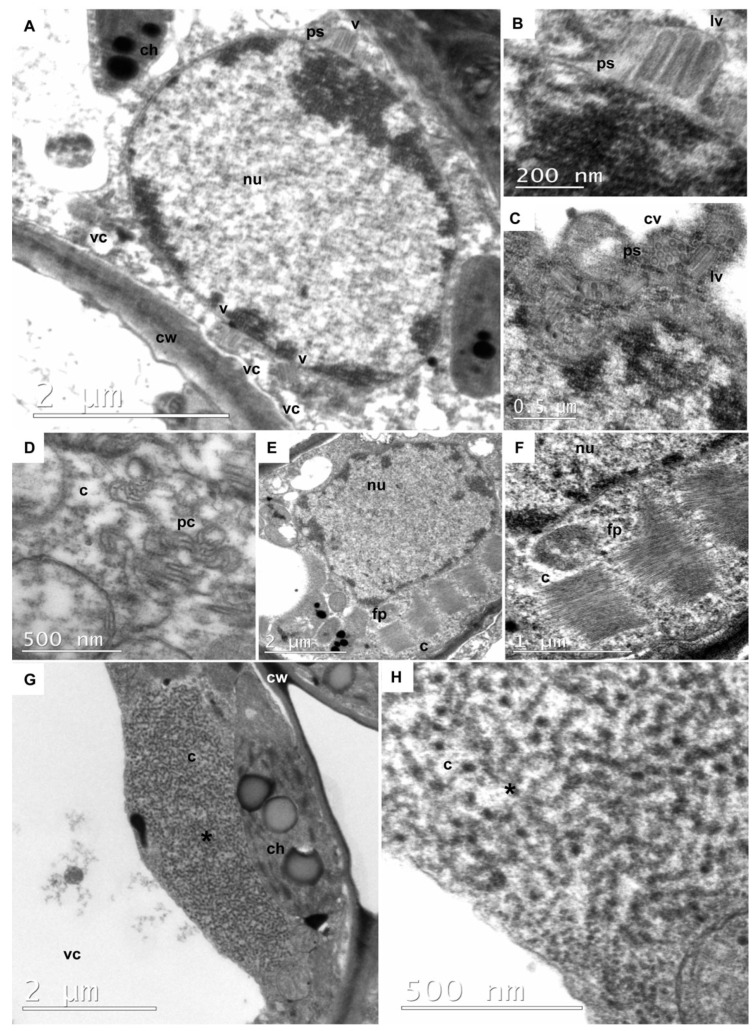
Transmission electron micrographs of ultrathin sections of symptomatic rhabdovirus-infected leaves of *P. gardneri* (Sample 1626) (**A**–**F**) and *P. riparia* (Sample 1624) (**G**,**H**). Sample 1626: (**A**) A low magnification image of a parenchyma cell showing pockets of perinuclear space (ps) containing bacilliform particles (v). (**B**) Detail of rhabdovirus particles sectioned longitudinally (lv) in the pockets perinuclear space (ps). (**C**) Detail of rhabdovirus particles in the pockets of perinuclear space (ps), cross sectioned (cv), revealing the outer membrane and the tubular inner component (nucleocapsid), as well as some of them sectioned longitudinally (lv). (**D**) Cross section of cylindrical inclusions, exhibiting pinwheel configuration (pc), in the cytoplasm (c) of a parenchymal cell, indicative of co-infection by potyvirus, possibly cowpea aphid-borne mosaic virus (CABMV). (**E**,**F**) Cytoplasmic inclusions of aggregates of elongated and flexuous particles (fp) in the cytoplasm (c) of the parenchymal cell. They possibly represent masses of particles of the carlavirus CPMMV. Sample 1626: (**G**,**H**) Overview of a viroplasm (*) formed by coiled filamentous material, possibly naked viral ribonucleoprotein, in the cytoplasm (c) of a parenchymal cell. Other cellular structures can be observed, including a vacuole (vc), a chloroplast (ch), a nucleus (nu), and a cell wall (cw).

Transmission electron microscopy (TEM) revealed the presence of rhabdovirus-like particles in tissues of *Passiflora* accessions from the BAG-PF collection (Figure 6). Selected samples that tested positive for rhabdoviruses by RT-PCR were further analyzed using TEM.

In sample 1626 (*P. gardneri*) (Figure 6A–F), bacilliform particles were identified within the cell nucleus, a characteristic feature of *Alphanucleorhabdovirus* (Figure 6A–C) [74]. A viroplasm was observed in the nucleus (Figure 6A). In the perinuclear space, longitudinal bacilliform particles (Figure 6B,C) and cross-sectioned particles (Figure 6C) exhibited a distinct outer membrane and a tubular inner component consistent with a nucleocapsid. Additionally, other structures were also identified in sample 1626 cells, such as pinwheel inclusions typical of potyvirus-induced cytopathology (Figure 6D), and cytoplasmic inclusions of aggregates of elongated and flexuous particles, resembling carlavirus structures (Figure 6E,F). These findings support the results obtained from the RT-PCR analyses (Table S1). Sample 1626 was confirmed to be infected with the potyvirus CABMV, the carlavirus CPMMV, the betacytorhabdovirus BaCV, and the alphanucleorhabdovirus PaNV2. Although BaCV was identified in this plant, no evidence of betacytorhabdovirus was observed in the TEM analysis.

No particles were visualized in sample 1624 (*P. riparia*) (Figure 6G,H). However, a coiled filamentous material resembling a viroplasm was observed in the cytoplasm. A similar electron-lucent viroplasm has been previously reported in bean cells infected with BaCV [33]. Sample 1624 tested positive for PFCV (Table S1). While no viral particles were visualized by TEM, RT-PCR analysis also revealed the presence of CABMV, CPMMV, and PaNV2 in this sample. The apparent scarcity of virus particles in *Passiflora* tissues and the difficulty in locating them within leaf tissues suggest that gammacytorhabdovirus and alphanucleorhabdovirus may be present at low concentrations in passion fruit plants. Similar observations have been reported in bean plants infected with the betacytorhabdovirus BaCV [33].

**Figure 7:**
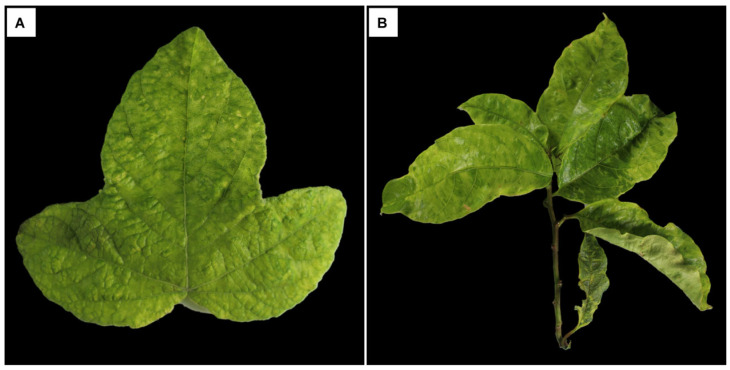
Symptoms displayed by *Passiflora* spp. leaves. (**A**) *P. gardneri* (Sample 1626) with light mosaic and (**B**) *P. riparia* (Sample 1624) showing leaf deformation, blistering, and mosaic.

The *Passiflora* plants examined by TEM exhibited mild mosaic symptoms (Figure 7A) along with leaf deformation, blistering, and mosaic (Figure 7B). It is important to emphasize that the symptoms observed in these two samples cannot be attributed to a single viral infection, as both plants were co-infected with at least four viruses, including the newly identified rhabdoviruses. This pattern was also observed in other samples analyzed in this study (Table S1) and corroborates findings from previous research [15].

There are very few documented cases of rhabdoviruses infecting passion fruit plants worldwide. Our findings are novel and significantly enhance the current understanding of the diversity of rhabdoviruses that infect plants, as well as the virome complexity within this important fruit crop in Brazil. Furthermore, the characterization of these newly identified rhabdoviruses, the development and availability of molecular diagnostic methods based on RT-PCR, and the identification of susceptible *Passiflora* species are essential for supporting the selection of virus-free plants in breeding programs.

## 4. Conclusions

This study expands the known range of viruses infecting *Passiflora* plants by identifying and characterizing three novel viruses: PFCV, PaNV1, and PaNV2. These rhabdoviruses were detected in various *Passiflora *species, but likely have a broader natural host range. PFCV was classified as a new member of the *Gammacytorhabdovirus* genus and is notable for having a glycoprotein, a unique feature among viruses in this genus. PaNV1 and PaNV2 are two novel members of the *Alphanucleorhabdovirus* genus. All three viruses were identified in mixed infections with at least one other virus. We observed in situ evidence of mixed infection, with PaNV2 particles found in the same tissues as a potyvirus and a carlavirus. This study also provides insight into the insect vectors responsible for the transmission and establishment of these viruses, as well as information regarding other viruses that have been previously identified only through transcriptome data. Further research is needed to evaluate the epidemiology of *Passiflora*-infecting rhabdoviruses and to assess the impacts of both single and mixed infections on crop productivity.

## Data Availability

Virus clones are available upon request. The sequences generated in this study are available at GenBank.

## Supplementary Materials

The following supporting information can be downloaded at: https://www.mdpi.com/article/10.3390/v17050725/s1, Figure S1. The strategy used for obtaining PFCV, PaNV1, and PaNV2 genomes; Figure S2. Genome organization of bean-associated cytorhabdovirus (BaCV-BAG3), cowpea mild mottle virus (CPMMV-BAG3), and cowpea aphid-borne mosaic virus (CABMV-BAG2) of passion fruit. CPMMV-BAG2 displaying conserved motifs highlighted in black boxes: methyltransferase (Met), C23 Peptidase (Pep_C23), RNA helicase (Hel1), and RNA-dependent RNA polymerase (RdRp). CABMV with mature proteolytic products highlighted in brown boxes P1, HC_Pro, P3, Pipo, 6K1, CI, 6K2, NIa_VPg, NIa_Pro, Nib, and CP.; Figure S3. Midpoint-rooted maximum likelihood phylogenetic tree inferred using an amino acid alignment of glycoprotein (G protein) of Passiflora cytorhabdovirus (PFCV-559; PFCV-29; PFCV-1630; PFCV-1591) and exemplar viruses for the species in the *Alphacytorhabdovirus* and *Betacytorhabdovirus* genera. The tree was inferred with 1000 ultrafast bootstrap replicates and the “find and apply best model” option. Only the values above 50% are shown. The viruses identified in this study are highlighted in yellow. All accession numbers used to construct the tree are listed in Supplementary Table S3 and Figure S4. Midpoint-rooted maximum likelihood phylogenetic tree inferred using an amino acid alignment of glycoprotein (G protein) of Passiflora nucleorhabdovirus 1 (PaNV1-B-564), and Passiflora nucleorhabdovirus 2 (PaNV2-1593) isolates, and exemplar viruses for the species in the *Alphanucleorhabdovirus*, *Betanucleorhabdovirus*, *Deltanucleorhabdovirus*, and *Gammanucleorhabdovirus* genera. The tree was inferred with 1000 ultrafast bootstrap replicates and the “find and apply best model” option. Only the values above 50% are shown. The viruses identified in this study are highlighted in yellow. All the virus sequence accession numbers used to construct the tree are listed in Supplementary Table S5 and Table S1. Detection of Passiflora cytorhabdovirus (PFCV), Passiflora nucleorhabdovirus 2 (PaNV2), Passiflora nucleorhabdovirus 1 (PaNV1), cowpea aphid-borne mosaic virus (CABMV), lettuce chlorosis virus (LCV), cowpea mild mottle virus (CPMMV), bean-associated cytorhabdovirus (BaCV), and cucurbit aphid-borne yellow virus (CABYV) in individual samples of *Passiflora* spp.; Table S2. Primers used in this study; Table S3. List of accession numbers of viruses used to construct the phylogenetic tree of *Alphacytorhabdovirus*, *Betacytorhabdovirus*, and *Gammacytorhabdovirus*; Table S4. Pairwise identity percentages between cognate genes of Passiflora cytorhabdovirus PFCV-1591 with PFCV-1630, PFCV-29, PFCV-559, and exemplar viruses for the species in the genus *Gammacytorhabdovirus*; Table S5. List of accession numbers of viruses used to construct phylogenetic tree of *Alphanucleorhabdovirus*, *Betanucleorhabdovirus*, *Gammanucleorhabdovirus*, and *Deltanucleorhabdovirus*; Table S6. Pairwise identity percentages between cognate genes of Passiflora nucleorhabdovirus 2 (PaNV2-1593 isolate) with Passiflora nucleorhabdovirus 1 (PaNV1-B-564) and exemplar viruses of the genus *Alphanucleorhabdovirus*; Table S7. Pairwise identity percentages between cognate genes of Passiflora nucleorhabdovirus 1 (PaNV1-B-564 isolate) with Passiflora nucleorhabdovirus 2 (PaNV2-1593) and exemplar viruses of the *Alphanucleorhabdovirus*.
