# Supplementary material for: Discovery and Genome Characterization of Three New Rhabdoviruses Infecting Passiflora spp. in Brazil
Source: Viruses. 2025 May 19;17(5):725. doi: 10.3390/v17050725 (PMC12116104; doi:10.3390/v17050725)
Supplement: Supplementary file 1 [file viruses-17-00725-s001.zip › viruses-3613989-Figures S1-S4.pdf]

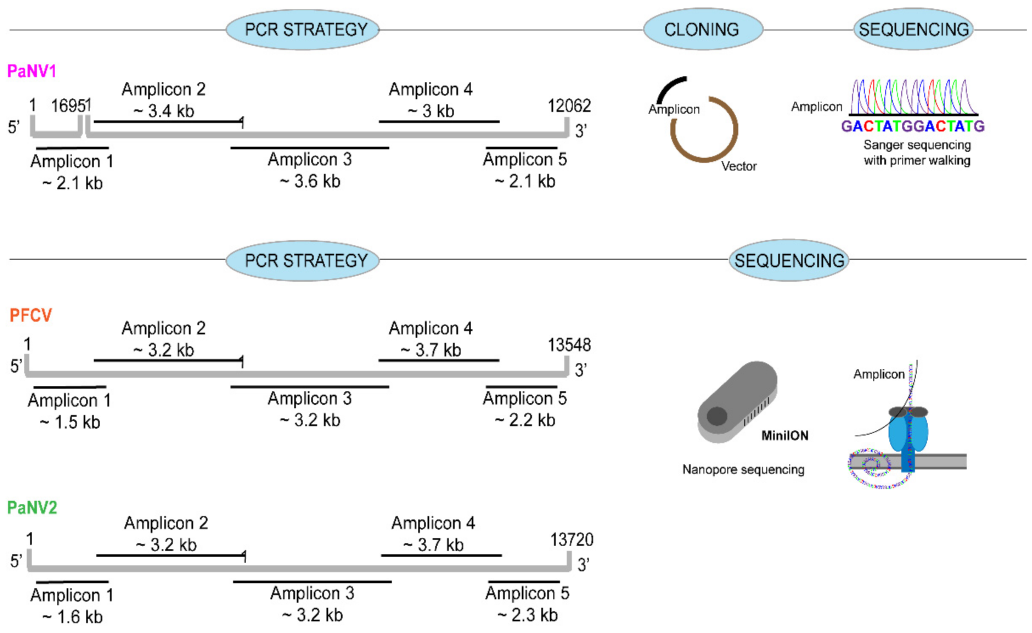

Figure S1. The strategy used for obtaining PFCV, PaNV1 and PaNV2 genomes.

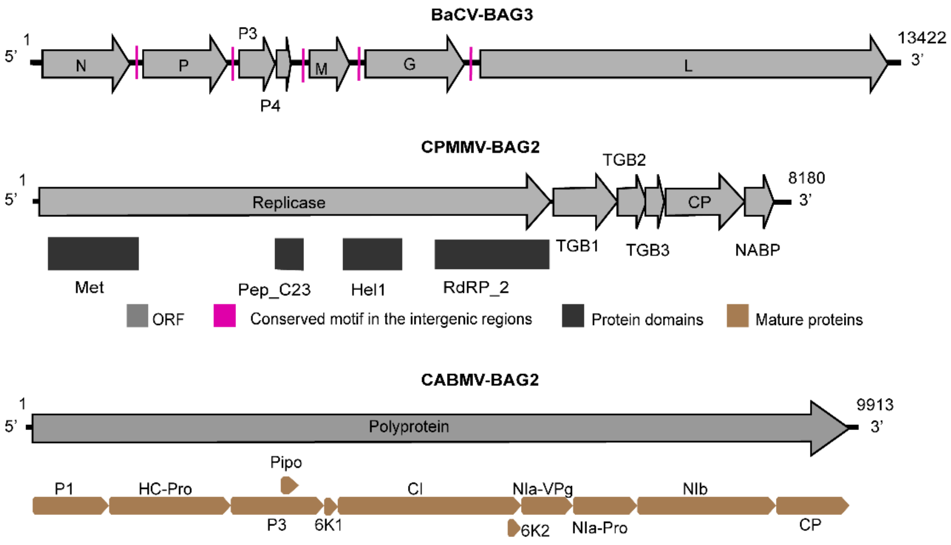

Figure S2. Genome organization of bean-associated cytorhabdovirus (BaCV-BAG3), cowpea mild mottle virus (CPMMV-BAG3) and cowpea aphid-borne mosaic virus (CABMV-BAG2) from passion fruit. CPMMV-BAG2 displaying conserved motifs highlighted in black boxes: methyltransferase (Met), C23 Peptidase (Pep_C23), RNA helicase (Hel1) and RNA-dependent RNA polymerase (RdRp). CABMV showing mature proteolytic products highlighted in brown box P1, HC_Pro, P3, Pipo, 6K1, CI, 6K2, NIa_VPg, NIa_Pro, Nib, and CP.

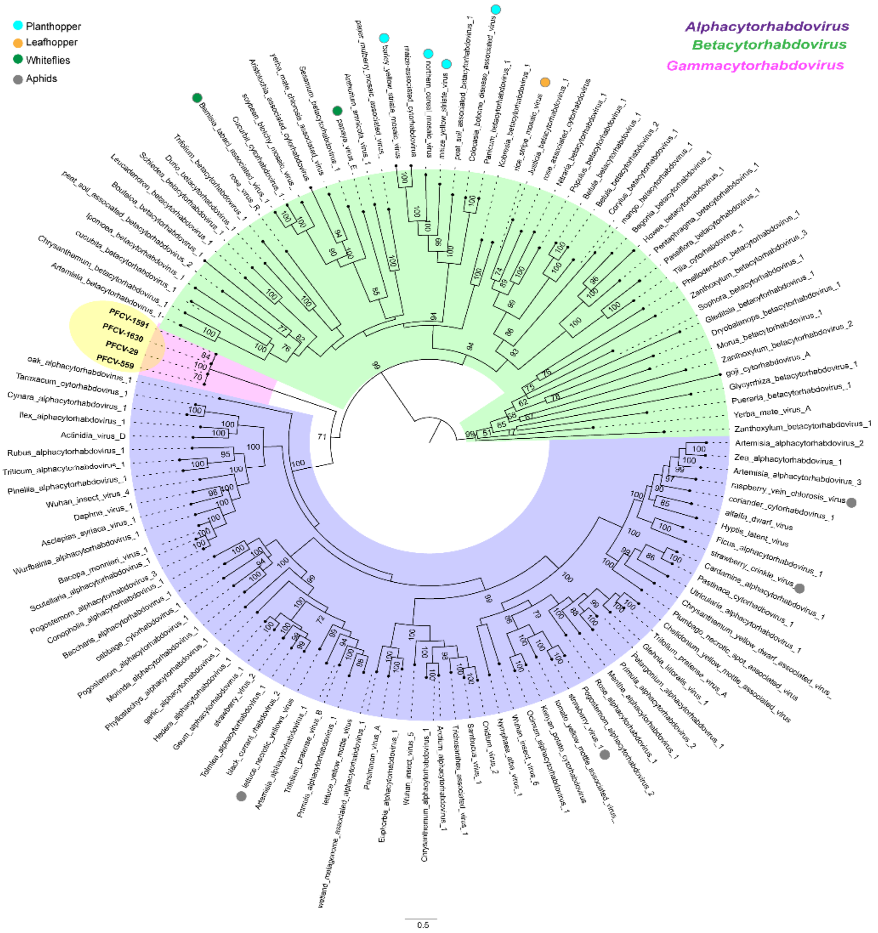

Figure S3. Midpoint-rooted maximum likelihood phylogenetic tree inferred using an amino acid alignment of the glycoprotein (G protein) of Passiflora cytorhabdovirus (PFCV-559; PFCV-29; PFCV-1630; PFCV-1591) and exemplar viruses for the species in the *Alphacytorhabdovirus* and *Betacytorhabdovirus* genera. The tree was inferred with 1000 ultrafast bootstrap replicates and the "find and apply best model" option. Only the values above 50% are shown. The viruses identified in this study are highlighted in yellow. All the accession numbers used to construct the tree are listed in Supplementary Table S3.

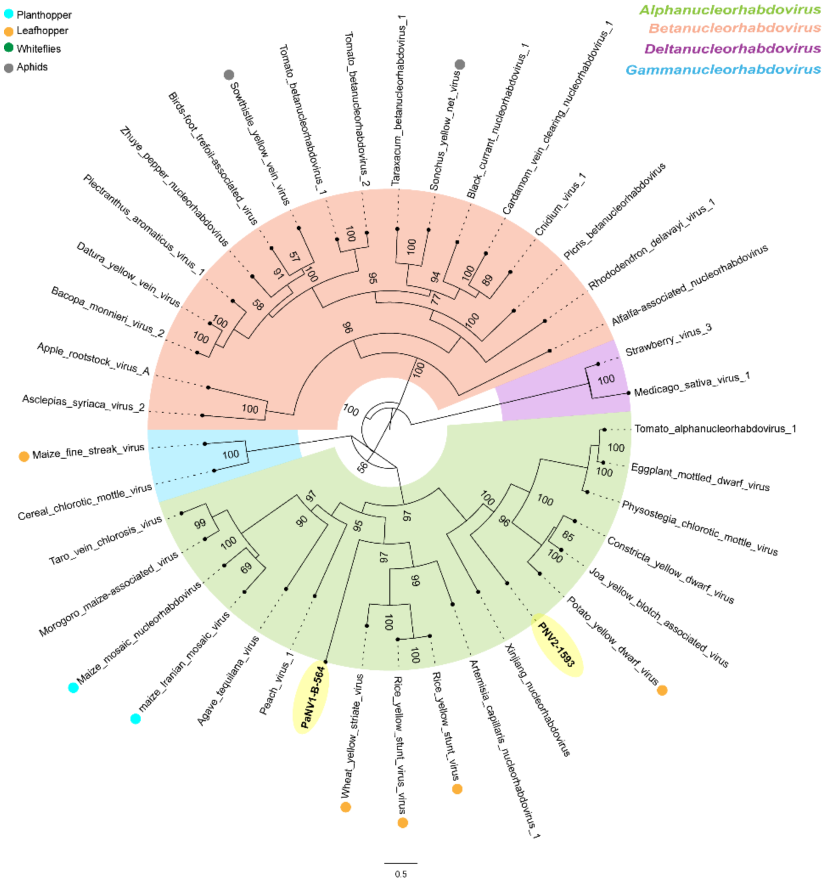

Figure S4. Midpoint-rooted maximum likelihood phylogenetic tree inferred using an amino acid alignment of the glycoprotein (G protein) of Passiflora nucleorhabdovirus 1 (PaNV1-B-564), and Passiflora nucleorhabdovirus 2 (PaNV2-1593) isolates, and exemplar viruses for the species in the *Alphanucleorhabdovirus*, *Betanucleorhabdovirus*, *Deltanucleorhabdovirus*, and *Gammanucleorhabdovirus* genera. The tree was inferred with 1000 ultrafast bootstrap replicates and the "find and apply best model" option. Only the values above 50% are shown. The viruses identified in this study are highlighted in yellow. All virus sequences accession numbers used to construct the tree are listed in Supplementary Table S5.
