# Supplementary material for: Discovery and Genome Characterization of Three New Rhabdoviruses Infecting Passiflora spp. in Brazil
Source: Viruses. 2025 May 19;17(5):725. doi: 10.3390/v17050725 (PMC12116104; doi:10.3390/v17050725)
Supplement: Supplementary file 1 [file viruses-17-00725-s001.zip › viruses-3613989-Tables S2-S6.pdf]

Table S2. Primers used in this study.

Target	Primer name	Sequence 5′- 3′	Gene/ Region (bp) TM	Reference
PFCV	PCV-BAG-N-182F	GCAGAACAGCATTCGTGATCA	N complete/ P partial (~1500) 60°C	
	PCV-BAG-N-1648R	CAGCCTTCTTTGCCTTATCGTT		
	BAG3-PCV7114F	ATGGACTGGGGCGAAGAGAG	L partial (~960) 61°C	
	BAG3-PCV8045R	GCGATATGTGCTGCGGATTGT		
PaNV1	PNV1-GL-7102R	GGAAGTGAAGTAGATACGACGAAAACTAC	L partial (1,352) 60°C	
	PNV1-L-5779F	CTTCTCAATGTCCTAAATAAGATGTATCTC		
PaNV2	BAG3-PNV4310F	ATGGATTACGAAGATTATGAAGACC	L partial (~1000) 61°C	
	BAG3-PNV5299R	CTAGCTGATCAACCGTCATG		
	BAG3-PNV-N-262F	CTTACTCGCTGTTGATATTTGAGCT	N complete /X partial (~1600) 60°C	
	BAG3-PNV-N-1890R	GACATTCTCCCGTTCCTAGCT		
PFCV-Amplicon 1	PCV-BAG-N-182F	GCAGAACAGCATTCGTGATCA	(~1500) 59°C	This study
	PCV-BAG-N-1648R	CAGCCTTCTTTGCCTTATCGTT		
PFCV-Amplicon 2	PCV-BAG-NPP3P4-679F	GTAGAAGATGAGGCGCCAACAAAAATAAAG	(~3200) 63°C	
	PCV-BAG-NPP3P4-3897R	TAGATCATACATATCGTCCGTAGTAGAGTC		
PFCV-Amplicon 3	PCV-BAG-P4MG-3660F	GATAGAATCTACTGGGCAGGGATGATACTG	(~3200) 63°C	
	PCV-BAG-P4MG-6880R	CAAATAAATTACCAACGTCCAGCACATTGG		
PFCV-Amplicon 4	PCV-BAG-P7L-6676F	CAGTTCTTGTAAGGAAATCGAGTATCAACG	(~3200) 63°C	
	PCV-BAG-P7L-9873R	GCCTATACATTCTTCTAGTACCTACTGATG		
PFCV-Amplicon 5	PCV-BAG-L-9664F	CTATGGCTACCATTCAAAGCAATTCTATGG	(~2200) 63°C	
	PCV-BAG-L-11873R	GATTACCAACCTCTTAGGCTCGGTTATATT		
PFCV-Amplicon 6	PCV_BAG_L_11666F	TATGAAAGTGTCACACTCAACCTTAACTGG	(~1700) 61°C	
	PCV_L_12968R	TGATTAGATAAGGGGTAGCGAGC		
PaNV1-Amplicon 1	PNV1-NP-119F	ACGCTCACCCTATGAACACAACAGAA	(~2300) 61°C	
	PNV1-NP-505R	TGATATTACGCCTGTTGGAAGATCTAG		
PaNV1-Amplicon 2	PNV1-PG-317F	AAGGTTTATTGCAGCGACCAAACTTG	(~3400) 61°C	
	PNV1-PG-3719R	CAATATGGTGAACAAGATTGAACGGAGTA		
PaNV1-Amplicon 3	PNV1-GL-3573F	GGTTAAGATGTCCTGAAAACCTATGTATC	(~3500) 61°C	
	PNV1-GL-7102R	GGAAGTGAAGTAGATACGACGAAAACTAC		
PaNV1-Amplicon 4	PNV1-L-6944F	GTTACCGATTTCCTATCTATCAGACATCC	(~3000) 61°C	
	PNV1-L-9943R	CCTCTTAAAGATGGTATAGATACTGGCATG		
PaNV1-Amplicon 5	PNV1-L-9668F	CCCGAATTTCTTGCAGGTATGTACTA	(~2100) 61°C	
	PNV1-L-11790R	CACATCACTATCAGCTTCACATTATTAC		
PaNV2-Amplicon 1	BAG3-PNV-N-262F	CTTACTCGCTGTTGATATTTGAGCT	(~1650) 60°C	
	BAG3-PNV-N-1890R	GACATTCTCCCGTTCCTAGCT		
PaNV2-	PNV-BAG-XPYM-1754F	CCGCCACAACCCACACTAGTATTAATAAA	(~3200)	

Amplicon 2		A	62°C	
	PNV-BAG-XPYM-4979R	GAGAAGGTTATCTCACCCAGGTTCTTC		
PaNV2-Amplicon 3	PNV-BAG-MGL-4777F	GACAAGAGAACAACCCACTATAATGAGTC	(~3200) 60°C	
	PNV-BAG-MGL-7952R	CTAGCTTTGATATGCGTGAGATGTC		
PaNV2-Amplicon 4	PNV-BAG-L-7767F	CTATTGAGGAGAAGAGGATTCGAGAGATTA	(~3700) 62°C	
	PNV-BAG-L-11471R	GAAAGTGTATAGTTTCATTCTTCCCTGCTC		
PaNV2-Amplicon 5	PNV-BAG-L-11273F	GTTACCAATACAGATCCTGAGTCTTTATGG	(~2300) 61°C	
	PNV-BAG-L-13577R	TTGTTATATCTCCATACTGACCTATCACCA		
3′Race cDNA	M10PacIT50VN	AAGCAGTGTTATCAACGCAGATTAATTAAT50VN	Tail and adaptor	Nicolini et al. [26]
.3′Race-GSP	M10	AAGCAGTGTTATCAACGCAGA	Adaptor	
3′Race-GSP PFCV	PCV_RACE3c_12839F	AGACATCAACAGCATACCTCCATT	3′ end PFCV (~700) 60°C	This study
3′Race-GSP PaNV1	PNV1_RACE3_11364F	CAGCTAAGAAGATCGTGTGGTTGG	3′ end PFCV (~600) 60°C	
3′Race-GSP PaNV2	PNV2_RACE3_13119F	GTACCTTAGACAAACTATCCGCAGT	3′ end PFCV (~500) 60°C	
PFCV	PCV_3end_R	ACTTAAATCTATTGACATGATTAGATAAGG	L partial/ 3′ UTR	
CABMV	CABMVLNJP2492F	GGTTCGTGATGTTTTGGTGCC	Partial HC-Pro/p3 genes (900) 60°C	Vidal et al. [20]
	CABMVLNJP3373R	CAAAAAGCACGCACTCACAAATC		
LCV	LCVRNA22793F	AAGGTTCAGATCCGTTCATCTTGTA	RNA2-Partial HSP70h/ p6.4/p60 genes (1199) 60°C	
	LCVRNA23997R	CTTCCACGCATTCTCTGAATAAGTC		
BaCV	BaCV-6491F	GAAGTCGCATAGCTCGTCGA	Partial G/L gene (678) 60°C	Alves-Freitas et al. [25]
	BaCV-7178R	GAGCGATAAGAACCTCCCCG		
CPMMV	CPMMVB22095F	AATCCTGGCTTTGAACTCGGA	Partial CP gene (457) 60°C	Vidal et al. [15]
	CPMMVB22535R	ATCGATCAGAGTATTTGAAGCCC		
	CPMMV-4000F	AACTTGGCCTTAGTGAACTCTACA	Partial RdRp (500) 58°C	Alves-Freitas et al. [25]
	CPMMV-4500R	ATTAGCTCTGTGCCTGGGGT		
CABYV	CE-9F	AATACGGTCGCGGCTAGAAATC	Partial CP/MP (600) 64°C	Kassen et al. [34]
	CE-10R	CTATTTCGGGTTCTGGACCTGGC		

GSP: Gene-Specific Primer

Table S3. List of accession numbers of viruses used to construct the phylogenetic tree of *Alphacytorhabdovirus*, *Betacytorhabdovirus*, and *Gammacytorhabdovirus*.

Virus specie	Virus name	Genbank acession number
Alphacytorhabdovirus actinidiae	Actinidia virus D	MW550041
Alphacytorhabdovirus allii	Garlic alphacytorhabdovirus 1	BK064275
Alphacytorhabdovirus alphaartemisiae	Artemisia alphacytorhabdovirus 1	BK064263
Alphacytorhabdovirus alphachysanthemi	Chrysanthemum yellow dwarf virus	MW039593
Alphacytorhabdovirus alphafragariae	Strawberry virus 1	MK211271
Alphacytorhabdovirus alphamedicagonis	Alfalfa dwarf virus	KP205452
Alphacytorhabdovirus alphapogostemi	Patchouli chlorosis-associated cytorhabdovirus	ON409991
Alphacytorhabdovirus alphaprimulae	Primula alphacytorhabdovirus 1	BK064294
Alphacytorhabdovirus alpharubi	Raspberry vein chlorosis virus	MK240091
Alphacytorhabdovirus alphatrifolii	Trifolium pratense virus A	MH982250
Alphacytorhabdovirus alphawuhaninsectum	Wuhan Insect virus 4	KM817650
Alphacytorhabdovirus arctii	Arctium alphacytorhabdovirus 1	BK064262
Alphacytorhabdovirus asclepiadis	Asclepias syriaca virus 1	BK014298
Alphacytorhabdovirus baccharis	Baccharis alphacytorhabdovirus 1	BK064266
Alphacytorhabdovirus bacopae	Bacopa monnieri virus 1	BK014479
Alphacytorhabdovirus betaartemisiae	Artemisia alphacytorhabdovirus 2	BK064264
Alphacytorhabdovirus betachrysanthemi	Chrysanthemum alphacytorhabdovirus 1	BK064269
Alphacytorhabdovirus betafragariae	Strawberry virus 2	MW480851
Alphacytorhabdovirus betamedicagonis	Medicago alphacytorhabdovirus 1	BK064279
Alphacytorhabdovirus betapogostemi	Pogostemom alphacytorhabdovirus 1	BK064288
Alphacytorhabdovirus betaprimulae	Primula alphacytorhabdovirus 2	BK064295
Alphacytorhabdovirus betarubi	Rubus alphacytorhabdovirus 1	BK064297
Alphacytorhabdovirus betatrifolii	Trifolium pratense virus B	MH982249
Alphacytorhabdovirus betawuhaninsectum	Wuhan Insect virus 5	KM817651
Alphacytorhabdovirus brassicicolae	Cabbage cytorhabdovirus 1	KY810772
Alphacytorhabdovirus cardaminis	Cardamine alphacytorhabdovirus 1	BK064267
Alphacytorhabdovirus chelidonii	Chelidonium yellow mottle associated virus	OR290114
Alphacytorhabdovirus cnidii	Cnidium virus 2	OQ442952
Alphacytorhabdovirus conopholis	Conopholis alphacytorhabdovirus 1	BK064270
Alphacytorhabdovirus coriandri	Coriander cytorhabdovirus 1	OR536958
Alphacytorhabdovirus cynarae	Cynara alphacytorhabdovirus 1	BK064271
Alphacytorhabdovirus daphnis	Daphne virus 1	OP180101
Alphacytorhabdovirus deltapogostemi	Pogostemom alphacytorhabdovirus 3	BK064292
Alphacytorhabdovirus euphorbiae	Euphorbia alphacytorhabdovirus 1	BK064272
Alphacytorhabdovirus fici	Ficus alphacytorhabdovirus 1	BK064274
Alphacytorhabdovirus fragariarugosus	Strawberry crinkle virus	MH129615
Alphacytorhabdovirus gammaartemisiae	Artemisia alphacytorhabdovirus 3	BK064265
Alphacytorhabdovirus gammapogostemi	Pogostemom alphacytorhabdovirus 2	BK064291
Alphacytorhabdovirus gammawuhaninsectus	Wuhan Insect virus 6	KM817652
Alphacytorhabdovirus gei	Geum alphacytorhabdovirus 1	BK064276
Alphacytorhabdovirus glehniae	Glehnia littoralis virus 1	BK014304
Alphacytorhabdovirus hederae	Hedera alphacytorhabdovirus 1	BK064277
Alphacytorhabdovirus hyptisis	Hyptis latent virus	ON073823
Alphacytorhabdovirus ilicis	Ilex alphacytorhabdovirus 1	BK064278

Alphacytorhabdovirus kenyatuberosum	Kenyan potato cytorhabdovirus	MN689395
Alphacytorhabdovirus lactucanecante	Lettuce necrotic yellows virus	AJ867584
Alphacytorhabdovirus lactumaculante	Lettuce yellow mottle virus	EF687738
Alphacytorhabdovirus lycopersici	Tomato yellow mottle-associated virus	KY075646
Alphacytorhabdovirus menthae	Mentha alphacytorhabdovirus 1	BK064280
Alphacytorhabdovirus morindae	Morinda alphacytorhabdovirus 1	BK064281
Alphacytorhabdovirus nymphaeae	Nymphaea alba virus 1	BK014307
Alphacytorhabdovirus ocimi	Ocimum alphacytorhabdovirus 1	BK064284
Alphacytorhabdovirus paludis	Wetland metagenome associated alphacytorhabdovirus 1	BK064302
Alphacytorhabdovirus pastinacae	Pastinaca cytorhabdovirus 1	OL472112
Alphacytorhabdovirus pelargonii	Pelargonium alphacytorhabdovirus 1	BK064285
Alphacytorhabdovirus persimmon	Persimmon virus A	AB735628
Alphacytorhabdovirus phyllostachysis	Phyllostachys alphacytorhabdovirus 1	BK064286
Alphacytorhabdovirus pinelliae	Pinellia alphacytorhabdovirus 1	BK064287
Alphacytorhabdovirus plumagonis	Plumbago necrotic spot associated virus	OR335651
Alphacytorhabdovirus querci	Oak alphacytorhabdovirus 1	BK064283
Alphacytorhabdovirus ribes	Black currant cytorhabdovirus 1	OP352885
Alphacytorhabdovirus rosae	Rose alphacytorhabdovirus 1	BK064296
Alphacytorhabdovirus sambuci	Sambucus cytorhabdovirus	OM523356
Alphacytorhabdovirus scutellariae	Scutellaria alphacytorhabdovirus 1	BK064298
Alphacytorhabdovirus taraxaci	Taraxacum cytorhabdovirus 1	OL472125
Alphacytorhabdovirus tolmieae	Tolmeia alphacytorhabdovirus 1	BK064299
Alphacytorhabdovirus trichonsathei	Trichosanthes associated rhabdovirus 1	BK011194
Alphacytorhabdovirus tritici	Triticum alphacytorhabdovirus 1	BK064300
Alphacytorhabdovirus utriculariae	Utricularia alphacytorhabdovirus 1	BK064301
Alphacytorhabdovirus wurfbainiae	Wurfbainia alphacytorhabdovirus 1	BK064303
Alphacytorhabdovirus zeae	Zea alphacytorhabdovirus 1	BK064304
Betacytorhabdovirus alphabetulae	Betula betacytorhabdovirus 1	BK064307
Betacytorhabdovirus alphacucurbitae	Cucurbit cytorhabdovirus 1	MT381995
Betacytorhabdovirus alpharosae	Rose virus R	MT952336
Betacytorhabdovirus alphaturbaesoli	Peat soil associated betacytorhabdovirus 1	BK064330
Betacytorhabdovirus alphazanthoxyli	Zanthoxilum betacytorhabdovirus 1	BK064342
Betacytorhabdovirus anthurii	Anthurium amnicola virus 1	BK014302
Betacytorhabdovirus aristolochiae	Aristolochia-associated cytorhabdovirus	OR090884
Betacytorhabdovirus artemisiae	Artemisia betacytorhabdovirus 1	BK064305
Betacytorhabdovirus begoniae	Begonia betacytorhabdovirus 1	BK064306
Betacytorhabdovirus bemisiae	Bemisia tabaci-associated virus 1	BK014303
Betacytorhabdovirus betabetulae	Betula betacytorhabdovirus 2	BK064308
Betacytorhabdovirus betacucurbitae	Cucurbita betacytorhabdovirus 1	BK064312
Betacytorhabdovirus betarosae	Rose associated cytorhabdovirus	ON762421
Betacytorhabdovirus betaturbaesoli	Peat soil associated betacytorhabdovirus 2	BK064331
Betacytorhabdovirus betazanthoxyli	Zanthoxilum betacytorhabdovirus 2	BK064343
Betacytorhabdovirus bouteloae	Bouteloa betacytorhabdovirus 1	BK064309
Betacytorhabdovirus broussonetiae	Paper mulberry mosaic associated virus	MN872813
Betacytorhabdovirus caricae	Papaya virus E	MH282832
Betacytorhabdovirus chrysanthemi	Chrysanthemum betacytorhabdovirus 1	BK064310
Betacytorhabdovirus colocasiae	Colocasia bobone disease-associated virus	KT381973
Betacytorhabdovirus coryli	Corylus betacytorhabdovirus 1	BK064311
Betacytorhabdovirus cypripedii	Cypripedium betacytorhabdovirus 1	BK064313

Betacytorhabdovirus dryobalanopis	Dryobalanops betacytorhabdovirus 1	BK064314
Betacytorhabdovirus durionis	Durio betacytorhabdovirus 1	BK064315
Betacytorhabdovirus flaviyerbamate	Yerba mate chlorosis-associated virus	KY366322
Betacytorhabdovirus gammazanthoxyli	Zanthoxilum betacytorhabdovirus 3	BK064344
Betacytorhabdovirus gleditsiae	Gleditsia betacytorhabdovirus 1	BK064316
Betacytorhabdovirus glycinis	Soybean blotchy mosaic virus	OM681518
Betacytorhabdovirus glycyrrhizae	Glycyrrhiza betacytorhabdovirus 1	BK064317
Betacytorhabdovirus graminae	Northern cereal mosaic virus	AB030277
Betacytorhabdovirus hepaticae	Hepatica betacytorhabdovirus 1	BK064318
Betacytorhabdovirus hordei	Barley yellow striate mosaic virus	KM213865
Betacytorhabdovirus howeae	Howea betacytorhabdovirus 1	BK064319
Betacytorhabdovirus ipomoeae	Ipomoea betacytorhabdovirus 1	BK064320
Betacytorhabdovirus ixeris	Ixeris denticulata-associated rhabdovirus	OQ927981
Betacytorhabdovirus justiciae	Justicia betacytorhabdovirus 1	BK064321
Betacytorhabdovirus kobresiae	Kobresia betacytorhabdovirus 1	BK064322
Betacytorhabdovirus leucadendri	Leucadendron betacytorhabdovirus 1	BK064323
Betacytorhabdovirus lycii	Goji cytorhabdovirus A	OR489165
Betacytorhabdovirus mangonis	Mango betacytorhabdovirus 1	BK064325
Betacytorhabdovirus maydis	Maize yellow striate virus	KY884303
Betacytorhabdovirus maysflavostriatis	Maize associated cytorhabdovirus	KY965147
Betacytorhabdovirus mori	Morus betacytorhabdovirus 1	BK064326
Betacytorhabdovirus nitrariae	Nitraria betacytorhabdovirus 1	BK064327
Betacytorhabdovirus oryzae	Rice stripe mosaic virus	KX525586
Betacytorhabdovirus panici	Panicum betacytorhabdovirus 1	BK064328
Betacytorhabdovirus passiflorae	Passiflora betacytorhabdovirus 1	BK064329
Betacytorhabdovirus pentaphragmae	Pentaphragma betacytorhabdovirus 1	BK064332
Betacytorhabdovirus phellodendri	Phellodendron betacytorhabdovirus 1	BK064333
Betacytorhabdovirus populi	Populus betacytorhabdovirus 1	BK064334
Betacytorhabdovirus puerariae	Pueraria betacytorhabdovirus 1	BK064335
Betacytorhabdovirus rudbeckiae	Rudbeckia virus 1	ON185810
Betacytorhabdovirus schiedeae	Schiedea betacytorhabdovirus 1	BK064338
Betacytorhabdovirus sesami	Sesamum betacytorhabdovirus 1	BK064336
Betacytorhabdovirus sophorae	Sophora betacytorhabdovirus 1	BK064339
Betacytorhabdovirus tagetis	Tagetes erecta virus 1	BK014308
Betacytorhabdovirus tiliae	Tilia cytorhabdovirus 1	OX411436
Betacytorhabdovirus trifolii	Trifolium betacytorhabdovirus 1	BK064340
Betacytorhabdovirus viciae	Vicia betacytorhabdovirus 1	BK064341
Betacytorhabdovirus yerbamate	Yerba mate virus A	MN781667
Gammacytorhabdovirus alphacuscutae	Cuscuta gammacytorhabdovirus 1	BK064349
Gammacytorhabdovirus alphafraxini	Fraxinus gammacytorhabdovirus 1	BK064353
Gammacytorhabdovirus apii	Celery gammacytorhabdovirus 1	BK064347
Gammacytorhabdovirus argyranthemi	Argyranthemum gammacytorhabdovirus 1	BK064345
Gammacytorhabdovirus betacuscutae	Cuscuta gammacytorhabdovirus 2	BK064350
Gammacytorhabdovirus betafraxini	Fraxinus gammacytorhabdovirus 2	BK064354
Gammacytorhabdovirus coptis	Coptis gammacytorhabdovirus 1	BK064348
Gammacytorhabdovirus cypripedii	Cypripedium gammacytorhabdovirus 1	BK064351
Gammacytorhabdovirus dauci	Carrot gammacytorhabdovirus 1	BK064346
Gammacytorhabdovirus epipactis	Epipactis gammacytorhabdovirus 1	BK064352
Gammacytorhabdovirus gymnadeniae	Gymnadenia densiflora virus 1	BK014305
Gammacytorhabdovirus heliospermae	Heliosperma gammacytorhabdovirus 1	BK064355

Gammacytorhabdovirus hibisci	Hibiscus gammacytorhabdovirus 1	BK064356
Gammacytorhabdovirus lonatis	Lonas gammacytorhabdovirus 1	BK064357
Gammacytorhabdovirus lupinis	Lupinus gammacytorhabdovirus 1	BK064358
Gammacytorhabdovirus rhopalocnemis	Rhopalocnemis gammacytorhabdovirus 1	BK064359
Gammacytorhabdovirus silenis	Silene gammacytorhabdovirus 1	BK064360
Gammacytorhabdovirus trachyspermi	Trachyspermum ammi virus 1	BK014309

Table S4. Pairwise identity percentages between cognate genes of Passiflora cytorhabdovirus PFCV-1591 with PFCV-1630, PFCV-29, PFCV-559 and exemplar viruses for the species in the genus *Gammacytorhabdovirus*.

	PFCV-1591					
**Virus**	**Full genome nt**	**N nt/aa**	**P nt/aa**	**G nt/aa**	**M nt/aa**	**L nt/aa**
PFCV-1630	99.7	99.7/99.3	99.9/100.0	99.4/99.1	99.8/100.0	99.6/99.4
PFCV-29	99.3	99.4/99.1	99.0/99.0	99.5/99.5	99.5/99.0	99.4/99.0
PFCV-559	99.6	99.4/99.1	99.7/100.0	99.5/99.6	99.5/99.0	99.6/99.7
Argyranthemum gammacytorhabdovirus 1	38.1	41.0/48.4	38.9/27.2	nd	32.2/25.1	38.4/49.3
Carrot gammacytorhabdovirus 1	39.3	36.7/38.6	39.6/22.4	nd	34.8/23.3	39.8/50.3
Celery gammacytorhabdovirus 1	38.6	38.7/42.3	40.4/28.9	nd	31.0/25.1	39.0/50.5
Coptis gammacytorhabdovirus 1	38.7	39.6/49.9	36.5/30.5	nd	31.4/25.9	39.2/51.2
Cuscuta gammacytorhabdovirus 1	39.5	42.3/47.6	40.1/34.5	nd	36.2/25.5	38.4/50.4
Cuscuta gammacytorhabdovirus 2	40.1	43.9/47.6	38.9/34.0	nd	35.8/27.0	38.9/51.0
Cypripedium gammacytorhabdovirus 1	38.6	41.5/50.0	38.3/28.2	nd	35.1/26.0	39.2/49.7
Epipactis gammacytorhabdovirus 1	38.6	39.7/47.7	39.9/33.0	nd	nd	38.6/50.5
Fraxinus gammacytorhabdovirus 1	40.4	41.7/53.3	37.5/29.3	nd	36.8/38.6	40.1/49.6
Fraxinus gammacytorhabdovirus 2	39.7	42.9/51.9	37.8/29.6	nd	33.4/34.7	39.0/49.8
Gymnadenia densiflora virus 1	39.5	38.7/45.2	39.0/31.4	nd	35.8/28.8	39.0/50.2
Heliosperma gammacytorhabdovirus 1	38.3	38.5/44.0	35.6/29.3	nd	36.7/25.8	38.3/49.5
Hibiscus gammacytorhabdovirus 1	39.3	38.1/41.2	41.7/22.4	nd	33.5/26.6	39.3/50.3
Lonas gammacytorhabdovirus 1	38.1	40.0/49.1	37.0/25.8	nd	33.1/27.4	38.3/49.6
Lupinus gammacytorhabdovirus 1	39.4	38.5/48.4	39.0/27.5	nd	36.4/27.3	40.1/51.5
Rhopalocnemis gammacytorhabdovirus 1	39.3	39.0/48.5	40.5/33.3	nd	nd/nd	38.4/49.3
Silene gammacytorhabdovirus 1	38.0	38.0/43.3	39.5/31.5	nd	30.6/30.4	37.9/49.2
Trachyspermum ammi virus 1	39.0	37.8/40.4	35.5/31.6	nd	34.4/25.3	39.4/50.0

N: nucleoprotein, P: phosphoprotein, M: matrix protein, G: glycoprotein, L: and RNA-dependent RNA polymerase – RdRP. nd= not determined. nt: nucleotides, aa: amino acids. All the accession numbers for the gammacytorhabdoviruses are listed in Table S3.

Table S5. List of accession numbers of viruses used to construct phylogenetic tree of *Alphanucleorhabdovirus*, *Betanucleorhabdovirus*, *Gammanucleorhabdovirus*, and *Deltanucleorhabdovirus*

Virus specie	Virus name	Genbank acession number
Alphanucleorhabdovirus agavis	Agave tequilana virus 1	BK014297
Alphanucleorhabdovirus artemisiae	Artemisia capillaris nucleorhabdovirus 1	OM372677
Alphanucleorhabdovirus colocasiae	Taro vein chlorosis virus	AY674964
Alphanucleorhabdovirus constrictae	Constricta yellow dwarf virus	KY549567
Alphanucleorhabdovirus joa	Joa yellow blotch associated virus	MW014292
Alphanucleorhabdovirus lycopersici	Tomato alphanucleorhabdovirus 1	OL472126
Alphanucleorhabdovirus maydis	Maize mosaic virus	AY618418
Alphanucleorhabdovirus melongenae	Eggplant mottled dwarf virus	KJ082087
Alphanucleorhabdovirus morogoromaydis	Morogoro maize-associated virus	MK063878
Alphanucleorhabdovirus oryzae	Rice yellow stunt virus	AB011257
Alphanucleorhabdovirus oryzae	Rice yellow stunt virus viral	AB516283
Alphanucleorhabdovirus physostegiae	Physostegia chlorotic mottle virus	KX636164
Alphanucleorhabdovirus pruni	Peach virus 1	MN520414
Alphanucleorhabdovirus tritici	Wheat yellow striate virus	MG604920
Alphanucleorhabdovirus tuberosum	Potato yellow dwarf virus	GU734660
Alphanucleorhabdovirus xinjianensis	Xinjiang alphanucleorhabdovirus	MW897039
Alphanucleorhabdovirus zeairanense	Maize Iranian mosaic virus	MF102281
Betanucleorhabdovirus alphalycopersici	Tomato betanucleorhabdovirus 1	OL472119
Betanucleorhabdovirus asclepiadis	Asclepias syriaca virus 2	BK014299
Betanucleorhabdovirus bacopae	Bacopa monnieri virus 2	BK014480
Betanucleorhabdovirus betalycopersici	Tomato betanucleorhabdovirus 2	OL472114
Betanucleorhabdovirus cardamomi	Cardamom vein clearing virus	MN273311
Betanucleorhabdovirus cnidii	Cnidium virus 1	MZ983390
Betanucleorhabdovirus daturae	Datura yellow vein virus	KM823531
Betanucleorhabdovirus loti	Birds-foot trefoil-associated virus	BK010826
Betanucleorhabdovirus mali	Apple rootstock virus A	MH778545
Betanucleorhabdovirus medicagonis	Alfalfa-associated nucleorhabdovirus	MG948563
Betanucleorhabdovirus picridis	Picris betanucleorhabdvirus 1	OL472117
Betanucleorhabdovirus plectranthi	Plectranthus aromaticus virus 1	BK014300
Betanucleorhabdovirus retesonchi	Sonchus yellow net virus	L32603
Betanucleorhabdovirus rhododendri	Rhododendon delavayi virus 1	BK014301

Betanucleorhabdovirus ribes	Blackcurrant-associated rhabdovirus	MF543022
Betanucleorhabdovirus taraxi	Taraxacum betanucleorhabdvirus 1	OL472118
Betanucleorhabdovirus venasonchi	Sowthistle yellow vein virus	MT185675
Betanucleorhabdovirus zanthoxyli	Zhuye pepper nucleorhabdovirus	MH323437
Deltanucleorhabdovirus fragariae	Strawberry virus 3	MW503935
Deltanucleorhabdovirus medicagonis	Medicago sativa virus 1	ON246246
Gammanucleorhabdovirus cerealis	Cereal chlorotic mottle virus	MW731536
Gammanucleorhabdovirus maydis	Maize fine streak virus	AY618417

Table S6. Pairwise identity percentages between cognate genes of Passiflora nucleorhabdovirus 2 (PaNV2-1593 isolate) with Passiflora nucleorhabdovirus 1 (PaNV1-B-564) and exemplar viruses of the genus *Alphanucleorhabdovirus*.

	**PaNV2-1593**					
**Virus**	**Full genome nt**	**N nt/aa**	**P nt/aa**	**M nt/aa**	**G nt/aa**	**L nt/aa**
PaNV1-B-564	51.5	51.2/30.8	52.0/18.6	50.9/28.3	51.9/25.2	52.1/35.5
Agave tequilana virus 1	37.1	41.8/26.2	40.7/25.4	39.2/22.7	35.8/24.4	38.8/36.6
Artemisia capillaris nucleorhabdovirus 1	35.3	39.2/25.1	39.5/18.9	38.4/22.9	36.2/28.4	37.4/35.9
Taro vein chlorosis virus	36.6	40.1/27.4	43.6/22.8	39.4/16.7	38.7/29.7	37.4/35.9
Constricta yellow dwarf virus	37.6	43.4/40.8	40.9/26.0	39.0/22.7	38.8/36.2	41.8/46.1
Joa yellow blotch associated virus	36.6	42.6/40.5	43.0/19.4	38.3/21.1	36.3/38.4	41.3/46.3
Tomato alphanucleorhabdovirus 1	37.0	41.7/39.0	41.7/22.3	37.3/20.2	38.6/35.3	40.4/47.1
Maize mosaic nucleorhabdovirus	36.6	41.4/28.4	43.6/21.9	39.5/24.9	39.5/27.6	38.4/36.7
Eggplant mottled dwarf virus	36.9	42.4/40.0	41.3/20.8	39.0/18.8	38.8/36.0	40.7/47.1
Morogoro maize-associated virus	36.2	41.9/28.8	41.7/20.9	40.3/21.1	36.9/27.3	37.5/35.9
Rice yellow stunt virus	33.5	38.8/26.6	40.9/25.9	40.5/22.3	36.6/26.2	39.1/36.2
Rice yellow stunt virus viral	33.6	38.6/25.4	40.2/25.9	39.4/20.7	36.8/26.4	38.1/37.1
Physostegia chlorotic mottle virus	37.5	41.8/37.3	39.5/20.3	38.0/13.8	37.3/35.4	41.3/46.6
Peach virus 1	32.6	40.0/27.0	40.1/24.9	37.9/24.4	36.6/27.6	37.3/35.9
Wheat yellow striate virus	34.5	42.0/25.8	42.0/23.3	41.0/19.1	37.9/26.0	39.0/37.1
Potato yellow dwarf virus	36.2	42.7/39.7	41.8/19.7	39.1/20.6	37.2/37.4	40.7/45.8
Xinjiang nucleorhabdovirus	37.1	43.7/33.8	42.1/23.6	38.8/22.7	36.9/35.3	41.8/46.1
Maize Iranian mosaic virus	37.0	42.6/24.9	41.9/25.2	40.1/26.0	38.7/26.6	38.5/35.7

N: nucleoprotein, P: phosphoprotein, M: matrix protein, G: glycoprotein, L: and RNA-dependent RNA polymerase – RdRP. nd= not determined. nt: nucleotides, aa: amino acids. All the accession numbers for the alphanucleorhabdoviruses are listed in Table S5

Table S7. Pairwise identity percentages between cognates genes of Passiflora nucleorhabdovirus 1 (PaNV1-B-564 isolate) with Passiflora nucleorhabdovirus 2 (PaNV2-1593) and exemplar viruses of the *Alphanucleorhabdovirus*.

	PaNV1-B-564					
**Virus**	**Full genome nt**	**N nt/aa**	**P nt/aa**	**M nt/aa**	**G nt/aa**	**L nt/aa**
PaNV2-1593	51.5	51.2/30.8	52.0/18.6	50.9/28.3	51.9/25.2	52.1/35.5
Agave tequilana virus 1	39.4	38.4/27.4	41.5/24.7	40.6/22.4	35.6/25.7	36.1/33.1
Artemisia capillaris nucleorhabdovirus 1	35.7	38.3/37.2	38.6/21.7	36.1/23.6	34.6/33.6	37.9/41.8
Taro vein chlorosis virus	38.6	38.1/29.2	38.4/21.8	37.2/25.5	35.0/28.3	36.7/33.8
Constricta yellow dwarf virus	41.3	41.4/29.6	39.3/25.7	40.6/24.6	33.0/27.9	37.3/36.2
Joa yellow blotch associated virus	40.8	38.6/30.6	39.5/23.8	38.4/22.4	33.4/26.6	36.8/36.4
Tomato alphanucleorhabdovirus 1	40.2	37.8/28.4	39.3/26.9	38.4/24.7	35.7/27.4	36.9/37.6
Maize mosaic nucleorhabdovirus	39.3	39.1/26.0	38.0/27.6	36.9/20.4	35.3/28.6	36.3/32.6
Eggplant mottled dwarf virus	40.6	38.2/28.9	39.6/23.1	36.7/25.8	34.5/25.0	37.4/36.9
Morogoro maize-associated virus	39.0	39.2/28.5	36.2/27.2	36.6/23.3	33.3/29.6	36.3/33.7
Rice yellow stunt virus	32.1	40.4/34.8	37.6/22.4	36.2/29.3	37.8/27.9	37.5/40.9
Rice yellow stunt virus viral	32.1	38.8/36.0	38.9/23.2	36.2/29.3	37.7/27.1	37.5/41.1
Physostegia chlorotic mottle virus	40.6	39.1/29.8	40.6/23.3	38.9/26.9	33.2/26.1	38.3/36.2
Peach virus 1	34.7	39.8/32.4	39.9/24.0	35.5/19.0	33.8/26.9	36.0/34.4
Wheat yellow striate virus	31.7	39.2/34.9	39.6/24.0	34.8/24.1	35.8/28.4	38.6/41.2
Potato yellow dwarf virus	40.2	38.1/28.5	42.2/24.3	41.7/19.1	32.6/24.7	36.0/34.9
Xinjiang nucleorhabdovirus	40.9	40.4/31.1	43.2/23.6	40.4/21.3	34.9/25.4	36.7/35.8
Maize Iranian mosaic virus	39.5	37.7/25.0	40.8/21.9	38.8/20.9	34.2/27.9	37.3/34.9

N: nucleoprotein, P: phosphoprotein, M: matrix protein, G: glycoprotein, L: and RNA-dependent RNA polymerase – RdRP. nd= not determined. nt: nucleotides, aa: amino acids. All the accession numbers for the alphanucleorhabdoviruses are listed in Table S5.
